# OPTRACE: Optical Imaging–Guided Transplantation and Tracking of Cells in the Mouse Brain

**DOI:** 10.1002/advs.202514183

**Published:** 2025-12-12

**Authors:** Jinghui Wang, Honglin Tan, Colleen Russell, Mikolaj Walczak, Dawei Gao, Guanda Qiao, Xiaoxuan Fan, Chengyan Chu, Miroslaw Janowski, Piotr Walczak, Yajie Liang

**Affiliations:** ^1^ Department of Diagnostic Radiology and Nuclear Medicine University of Maryland School of Medicine Baltimore MD 21201 USA; ^2^ Department of Microbiology and Immunology University of Maryland School of Medicine Baltimore MD 21201 USA

**Keywords:** image‐guided transplantation, modeling cell injection, stem cell therapy, stroke, two‐photon fluorescence microscopy

## Abstract

Intracerebral cell transplantation holds promise for treating stroke and neurological disorders, yet challenges in precise delivery and post‐engraftment monitoring impede progress. This work introduces OPTRACE (OPtical imaging‐guided Transplantation and tRAcking of CElls), a two‐step optical framework integrating real‐time visualization during transplantation with longitudinal post‐transplantation in vivo cell tracking. Leveraging cost‐effective translucent glass micropipettes and two innovative predictive mathematical modeling—the retention–depth model (predicts retained fraction versus injection depth) and the hypoperfusion–volume model (predicts hypoperfused fraction versus graft volume)—that this work fits to data (depth–retention R^2^ = 0.91; volume–growth R^2^ = 0.78)—OPTRACE optimizes delivery parameters to maximize engraftment and minimize hypoxia. A novel pulse‐elevation injection technique further enhances the precision of superficial cortical retention. Following transplantation, multicolor labeling combined with two‐photon fluorescence microscopy permits longitudinal single‐cell tracking, revealing host microglial responses, and altered neuronal calcium signaling at the graft interface. OPTRACE provides micrometer precision, longitudinal dynamics and quantitative insights of cells during and after transplantation, accelerating mechanistic understanding and therapeutic development for regenerative cell therapies.

## Graphical abstract

OPTRACE establishes an optical, two‐step platform for intracerebral cell therapy. Transparent glass pipettes enable real‐time, image‐guided delivery, while multiplex genetic labeling with two‐photon and bioluminescence readouts supports longitudinal single‐cell tracking and host–graft dynamics. Predictive models tune depth and volume to maximize retention and limit hypoperfusion, unifying preparation, transplantation, and post‐engraftment analysis in an accessible workflow.

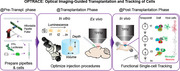

## 1 Introduction

Cell therapies are being actively explored, both preclinically and clinically, for the treatment of stroke, brain injury, and various neurodegenerative disorders^[^ ^1^, ^2^, ^3^, ^4^, ^5^, ^6^ ^]^ Recent advances in neurorestorative approaches emphasize stem cell technologies and biomaterials to overcome challenges in ischemic stroke.^[^ ^7^, ^8^ ^]^ However, the therapeutic potential of these cells depends critically on their precise and effective delivery.^[^ ^9^, ^10^ ^]^ Intracerebral transplantation typically involves injecting high‐density cell suspensions into damaged brain regions using a syringe‐attached needle. Phase I clinical trials have demonstrated the procedural safety of intracerebral cell implantation, with no significant adverse effects reported.^[^ ^11^, ^12^, ^13^ ^]^ Despite this, cell retention and viability remain low, with studies reporting survival rates of as little as 3%.^[^ ^9^, ^14^, ^15^, ^16^ ^]^ While post‐transplantation survival can be influenced by factors such as inflammation, lack of trophic support, or an unfavorable extracellular matrix, cell damage during injection and leakage from the injection site may also contribute to poor outcomes.^[^ ^9^, ^14^, ^16^ ^]^ Notably, there is limited guidance on predicting cell retention or growth outcomes based on injection depth or implantation volume during intracerebral cell delivery.

To improve cell retention during delivery, a critical step in successful cell transplantation is essential to closely monitor the injection process for optimizing delivery parameters accordingly. A major barrier to clinical translation has been the lack of noninvasive methods to assess whether appropriate cell doses are being administered.^[^ ^17^, ^18^ ^]^ Even in preclinical studies, reliable approaches for monitoring the cell delivery process remain limited.^[^ ^19^ ^]^ On the other hand, in the post‐transplantation phase, there remains a critical need for noninvasive imaging techniques capable of tracking the survival, engraftment, migration, and distribution of transplanted stem cells in vivo with high spatial and temporal resolution.^[^ ^20^, ^21^ ^]^ Most existing studies on stem cell therapy rely on histological analysis at a few discrete timepoints to assess outcomes,^[^ ^22^, ^23^, ^24^ ^]^ which fails to capture the dynamics of implanted cells, or interactions between graft and host cells. A central challenge in advancing cell transplantation therapy is the limited understanding of donor cell fate in living animals—specifically their morphology, function, and interactions with host neurons and glial cells over time. Without appropriate tools, this post‐transplantation phase remains a “black box” in need of illumination.

In this study, we present OPTRACE (OPtical imaging‐guided Transplantation and tRAcking of CElls), a two‐step framework leveraging optical imaging to overcome critical challenges in cell transplantation and post‐transplantation phases. During transplantation, OPTRACE utilizes transparent glass micropipettes as injectors for real‐time visualization of the injection process, enabling precise optimization of injection depth and volume, validated by theoretical modeling. Notably, OPTRACE introduces the pulse‐elevation technique that significantly improves cell retention in the superficial layers of the mouse cerebral cortex compared to conventional continuous injection methods. Post‐transplantation, OPTRACE employs intravital two‐photon fluorescence microscopy (TPFM) for high‐resolution, longitudinal tracking of grafted cells, and dynamic monitoring of host neuronal and microglial responses at the graft site. Collectively, these integrated capabilities establish OPTRACE as a robust platform for addressing key barriers in cell transplantation and advancing regenerative cell therapies.

## 2 Results

### 2.1 Illuminating Cell Therapy through OPTRACE

**Figure 1:**
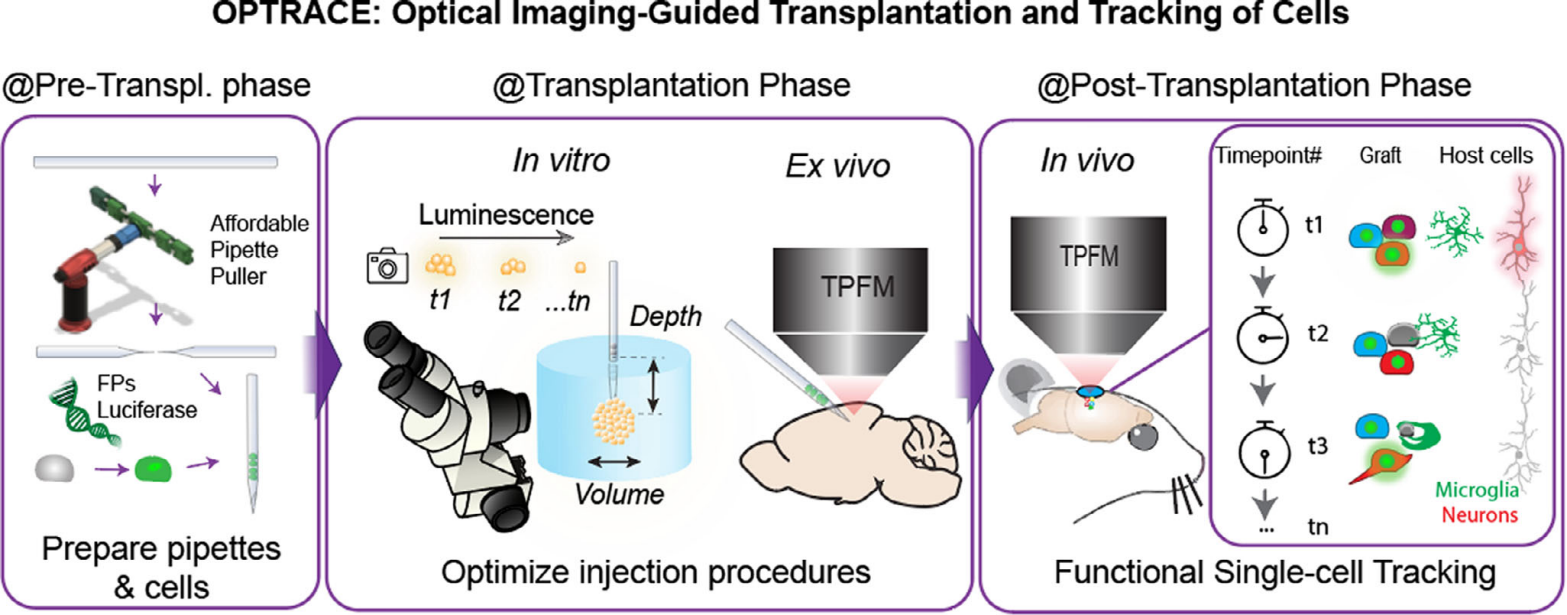
Schematic illustrating the application of OPTRACE (Optical Imaging‐Guided Transplantation and Tracking of Cells). During the pre‐transplantation phase, transparent glass pipettes are prepared for direct visualization of cells during loading and injection, and cells are genetically labeled with fluorescent reporters for quantification and tracking. In the transplantation phase, optical imaging enables real‐time monitoring of the procedure and assessment of cell retention and viability. Post‐transplantation, TPFM supports longitudinal tracking of implanted cells and reveals dynamic responses of host cells at the transplant site.

OPTRACE integrates three core components to advance intracerebral cell transplantation (**Figure** **1**). First, cost‐effective transparent glass pipettes enable direct visualization of cells during loading and injection, reducing procedural uncertainty. To enhance accessibility, we developed Affordable Pipette Pullers (APPs), a low‐cost method for fabricating functional injectors, eliminating reliance on expensive specialized equipment. Second, cells are genetically labeled with fluorescent proteins (FPs) or bioluminescent reporters (e.g., luciferase) for precise quantification or tracking. Multiplexed FPs assign unique spectral signatures for high‐fidelity single‐cell tracking, as shown in our prior work.^[^ ^25^ ^]^ These two components are prepared at the pre‐transplantation phase. The third component involves three optical imaging modalities: wide‐field microscopy, bioluminescence imaging, and TPFM. For the transplantation phase, wide‐field microscopy facilitates real‐time cell visualization, while bioluminescence imaging quantifies cell retention and monitors growth to validate theoretical models in vitro. As a powerful tool increasingly used for high‐resolution imaging of brain tissue,^[^ ^26^, ^27^, ^28^ ^]^ TPFM supports both transplantation procedure (real‐time confirmation of optimized delivery) and post‐transplantation phases (longitudinal tracking of implanted cells in vivo), owing to its deep tissue penetration and subcellular resolution. The following sections detail OPTRACE's implementation and its potential to revolutionize intracerebral cell transplantation.

### 2.2 Affordable Transparent Pipette Pulling and Real‐Time Visualization Platform for Cell Transplantation in OPTRACE

**Figure 2:**
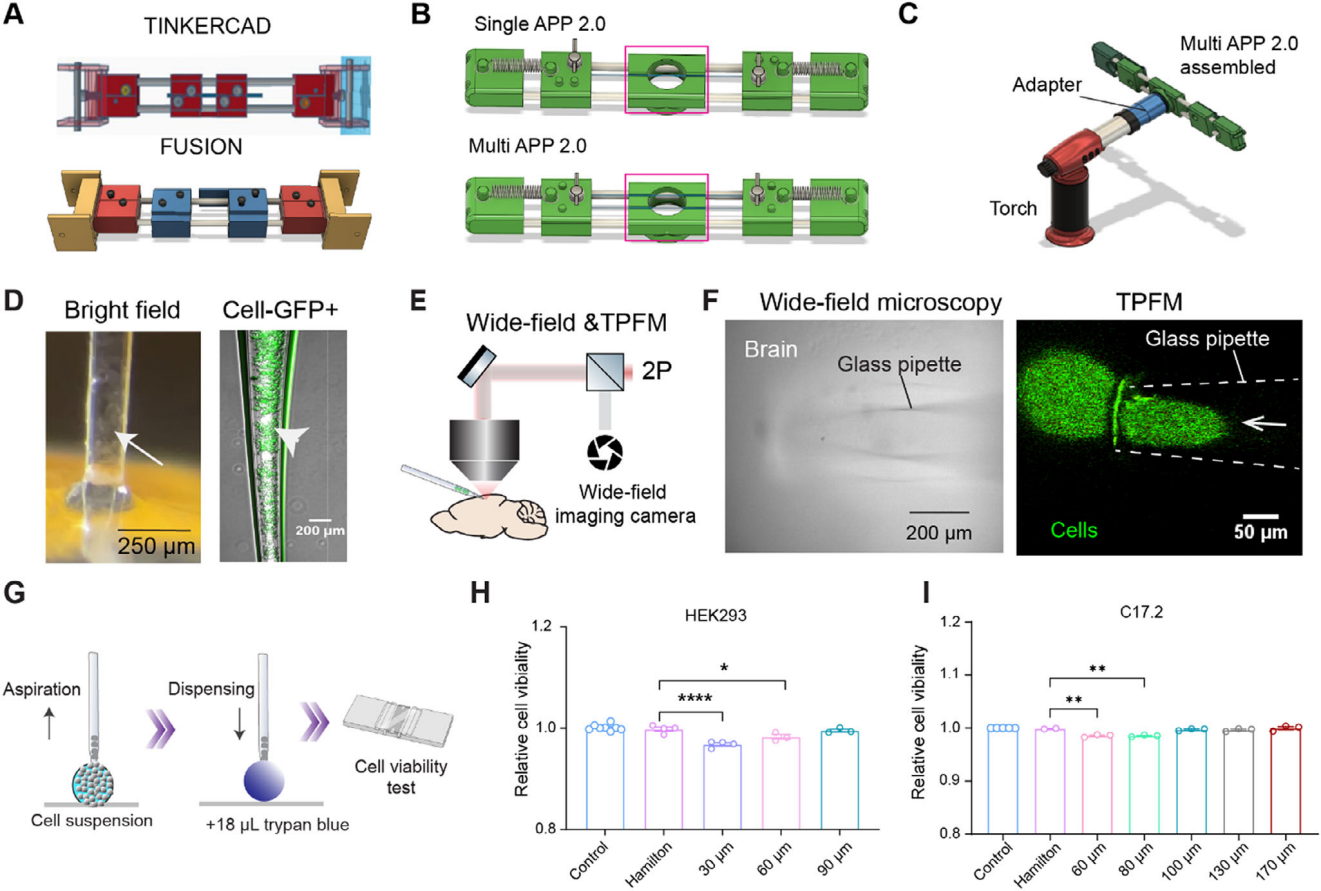
Upgraded APP to produce the injector and determination of optimal injector diameter for cells. A) APP designs in Tinkercad and in Fusion 360. B) Upgraded APP 2.0 includes single‐channel and multi‐channel designs. C) Assembled APP for use of pipette pulling. D) Bright field and fluorescence channels imaging of glass pipette filled with cells labeled with green fluorescent protein (GFP). E) Schematic of the ex vivo imaging setup. F) Wide‐field imaging (Left) and TPFM (Right) of a glass pipette filled with C17.2 cells labeled with GFP during injection into the mouse brain (ex vivo). G). Workflow for testing the effect of flowing through the needle on the viability of cells. H,I) Viability of HEK293 cells (H) or C17.2 (I) at different diameters of injectors. For (H): Hamilton 27G versus 30 µm: *p* = 0.0014; Hamilton 27G versus 60 µm: *p* = 0.0179, *n* = 3,2,2,3,3. For (I): Hamilton 27G versus 60 µm: *p* = 0.0126; Hamilton 27G versus 80 µm: *p* = 0.0362; *n* = 7,3,3,3,3,3,3. Differences were analyzed by one‐way ANOVA followed by Tukey's test. (*p < 0.05, ** p < 0.01).

To achieve OPTRACE, we utilized transparent glass pipettes as a replacement of metal needles, which forbid visualization of cells during cell injection. To overcome the cost barrier that limits access to commercial pipette pullers, we upgraded previous Tinkercad designs of APP^[^ ^29^ ^]^ by revising them through Fusion 360 (Autodesk, **Figure** **2**A), enhancing both the robustness and consistency of the APP. The redesigned APP 2.0 features improved ergonomics, reliability, and ease of use (Figure 2B, upper panel). Additionally, we developed a multi‐channel APP 2.0 capable of simultaneously producing four pipettes for high‐throughput fabrication (Figure 2B, lower panel). More details of APP2.0 designs are illustrated in Figure S1, Supporting Information. Design files are available at: https://github.com/liangy10/APP2. To improve usability, we also engineered a new torch adapter (Figure 2C) that securely attaches to the APP body via built‐in threads, significantly improving handling and precision during pipette pulling (Video S1, Supporting Information). Its effectiveness is demonstrated in imaging results (Figure 2D), where cells are visible within the glass pipette under both brightfield or fluorescence microscopy (indicated by arrows). More importantly, under both but not simultaneous wide‐field microscopy and TPFM (Figure 2E), we readily located pipettes (Figure 2F, left panel) containing fluorescently labeled cells and monitored the cell injection process into the mouse brain in real time under TPFM (Figure 2F, right panel, Video S2, Supporting Information).

Needle diameter plays a critical role in the success of delicate cell transplantation procedures.^[^ ^10^, ^30^, ^31^ ^]^ While small‐diameter needles are favored for minimizing tissue damage, they can compromise cell viability due to mechanical stress during injection. Thus, we examined the relationship between needle diameter and cell viability after flowing through the needle. Target cells (HEK293 as a control or mouse neural stem cell line C17.2) were aspirated and subsequently dispensed through the glass pipette needles (Figure 2G). Viability was evaluated, revealing significant reduction in the 30 µm (*p* = 0.0014) and 60 µm (*p* = 0.0179) groups compared to the Hamilton (27G size needle, inner diameter of 210 µm) group for HEK293 cells (Figure 2H), whereas the 90 µm pipette did not show a significant reduction (*p* = 0.7886, Figure 2H). For C17.2 cells, the 60 µm (*p* = 0.0126) and 80 µm pipettes (*p* = 0.0362) resulted in significantly reduced viability (Figure 2I), while no significant difference was observed for pipettes larger than 100 µm when compared to the Hamilton control (*p* = 0.328, Figure 2I). Therefore, diameters in the range of 100–120 µm were used for subsequent experiments.

### 2.3 Modeling Cell Retention Rate as a Function of Injection Depth for Optimizing Transplantation into the Brain

**Figure 3:**
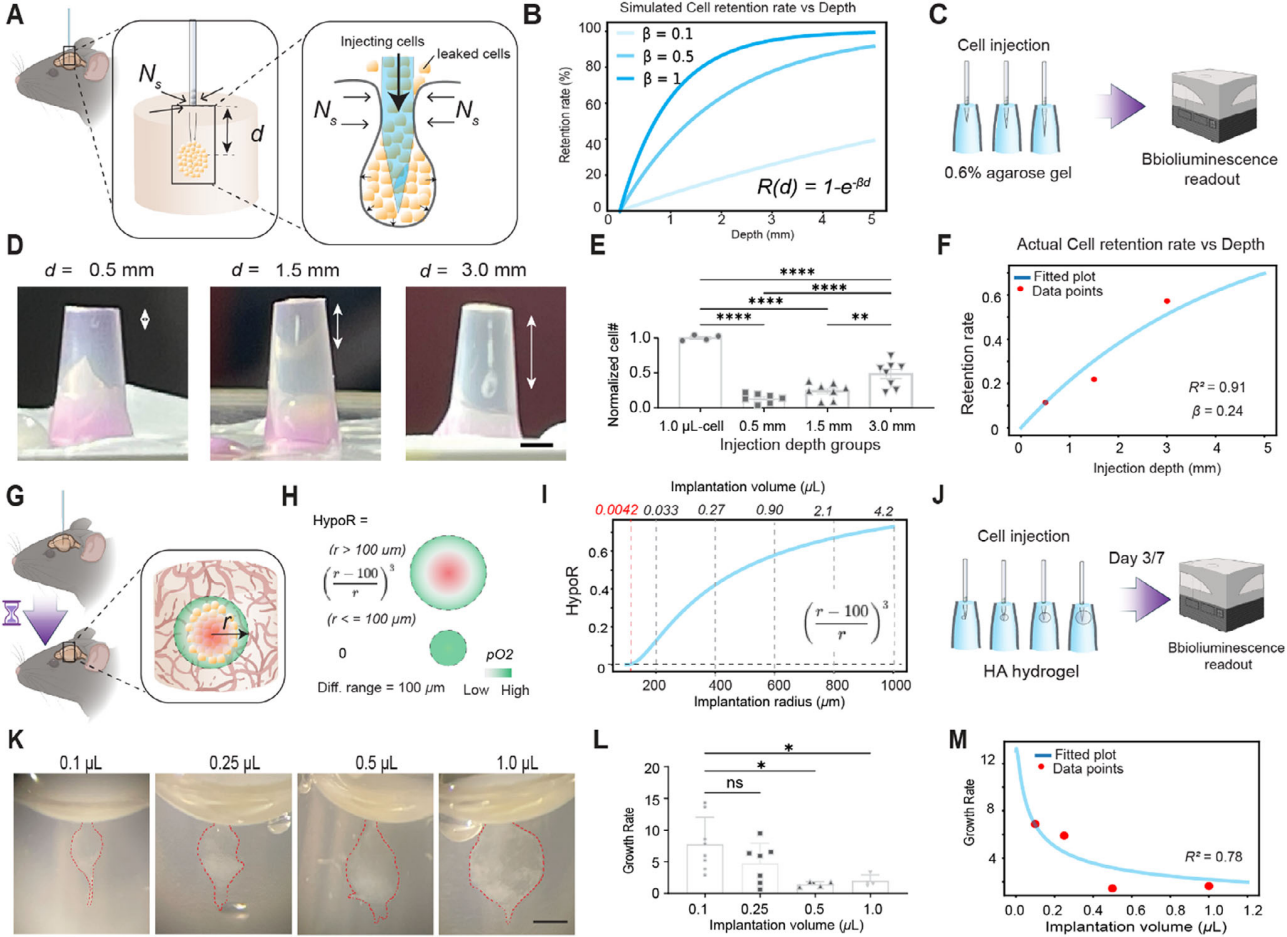
Modeling cell retention rate and hypoperfused cell fractions. A) Schematic representation of a needle inserted into the brain, where sealing force 𝑁𝑠 is proportional to needle insertion depth 𝑑. B) Simulated cell retention rate at different beta value. Experimental setup C) for validating the model after injecting cells at different depths within the 0.6% low‐melting agarose, D) using bioluminescence imaging to quantify retained cells (E, *p* < 0.0001, *n* = 4,7,8,8). F) Fitting the theoretical curve with experimental data. G) Schematic illustration of implanted cell mass undergoing hypoperfusion at post‐transplantation phase. H) Relationship between hypoperfused ratio and diameter of the implanted cell mass. I) HypoR as a function of radius of implanted cell mass. Upper horizontal axis shows corresponding volume of implanted cell mass. J) Experimental setup to verify the modeling. K) Cells of different volumes were injected into hydrogel. L) Quantification of growth rate after 4 days of culture. (0.5 µL, *p* = 0.0188), and 1.0 µL, *p* = 0.0343, *n* = 8,7,4,4). M) Fitting the theoretical curve with experimental data. Differences were analyzed by one‐way ANOVA followed by Tukey's test. (* *p* < 0.05, ** *p* < 0.01, **** *p <* 0.0001). Scale bar in (D) and (K), 1 mm.

Research indicates that the survival rate of transplanted neural progenitor cells can be as low as 1–2%, with substantial cell loss post‐transplantation.^[^ ^32^, ^33^, ^34^ ^]^ This highlights the importance of optimizing transplantation parameters. We modeled the relationship between the depth of injection (*d*) and the cell retention rate (*R*, **Figure** **3**A) with other fixed parameters, such as cell density, needle diameter, injection velocity, etc. Due to the viscosity of the brain tissue, there is a sealing vector (*N_s_*) pointing from the host tissue to the center of needle during the transplantation procedure. As more depth cells are injected inside the tissue (as *d* increases), there is more resistance against the cells preventing them from leaking out through the needle track. Therefore, the magnitude of this vector is in positive relation to the injection depth. For simplicity, assume it's a linear or power relationship:*N*(*d*) =  *k*· *d^n^*, where *k* > 0, *n* ≥ 1, in which n is adjustable to represent nonlinear growth. As retention rate *R* is positively related to sealing force, approaching 100%, we modeled it using a sigmoid or saturation function:*R*(*d*) = 100% • (1 − *e* ^−α*N*(*d*)^). Substituting N and assuming *n* = 1, we got *R*(*d*) = 100%  ×  •(1 − *e* ^−α*kd*^), where α is a scaling parameter related to how strongly sealing affects retention and *k* is the scaling coefficient for sealing force. If we merge the α and k into one constant *β*, we get R(d) = 100%  × • (1 − e^−βd^) or if in fraction,*R*(*d*) = 1 − *e* ^−β*d*^ (Equation 1, Figure 3B). This retention–depth model (Equation 1) intends to predict the increase in retention rate as a function of injection depth and reflect how sealing forces work in soft, viscoelastic tissue. At depth *d* = 0, *R(d)* = 0, meaning no retention as cells are all leaking out. As depth increases, retention approaches 100% asymptotically. The parameter 𝛽 controls how steeply retention increases with depth. A larger 𝛽 means we get close to 100% retention with shallower injections (Figure 3B), while a smaller 𝛽 means we need to inject deeper to achieve high retention. As this is a simplified function, we use 𝛽 as an integrated variable that is affected by tissue viscosity, expandability, engraft rigidity, and viscosity, needle shape, etc. But once transplantation settings are determined, such as target tissue, engraft, etc, 𝛽 could be determined through experiment, which was entailed below.

To obtain experimental support for our modeling, we injected mouse neural stem cells (C17.2) into different depths of the 0.6% low‐melting agarose gel (simulation of the brain tissue^[^ ^35^, ^36^ ^]^) and quantified the fraction of cells that are retained through bioluminescence imaging (BLI) as C17.2 cells express luciferase (Figure 3C). After injection, the needle tracks and cells were clearly visible (Figure 3D). Quantification of cell numbers in each group revealed that, compared to the 1.0 µL cell suspension group, signals from the 0.5, 1.5, and 3.0 mm depth groups were significantly reduced (Figure 3E and *p* < 0.0001). Further analysis revealed that the cell signal in the 3.0 mm group was significantly higher than in the 0.5 and 1.5 mm groups. However, there was no significant difference between the 0.5 mm and 1.5 mm groups (*p* = 0.4059, Figure 3E). We then used this data to calculate the 𝛽 value in the above Retention function and got the 𝛽 value of 0.24 with good fitting (Pear's square = 0.91, Figure 3F). Thus, under our experimental conditions, the equation *R*(*d*) = 1 − *e* ^−0.24*d*^ (Equation 2), serves as the retention rate prediction function. These findings confirm our modeling hypothesis and provide theoretical guidance for predicting cell retention rate as a function of injection depth.

### 2.4 Modeling Hypoperfused Cell Fractions as a Function of Graft Volume

After cells are implanted into the brain, they experience stress due to limited oxygen and nutrient delivery (Figure 3G), referred to as hypoperfusion.^[^ ^2^, ^37^ ^]^ Conceptually, the larger the implanted cell mass, the greater the fraction of cells that become hypoperfused. Under normal physiological conditions, the partial pressure of oxygen (pO_2_) in the cerebral cortex ranges from 30 to 40 mmHg. When pO_2_ drops below 5 mmHg, it is considered a state of critical hypoxia, which can cause irreversible damage within minutes if sustained.^[^ ^38^, ^39^ ^]^ Since the implanted cell mass lacks direct blood vessel perfusion for at least several days, cells located away from the periphery of the graft—and therefore farther from the host's well‐perfused brain tissue—are more prone to hypoxia. A mono‐exponential decay model is commonly used to approximate oxygen diffusion in tissue:^[^ ^40^, ^41^, ^42^ ^]^ *pO*2(*L*) = *pO*2*cap*· *e* ^−*L*/λ^ (Figure S2, Supporting Information), where L is the distance from the nearest capillary, *pO_2_cap* is the initial oxygen partial pressure near the capillary (typically assumed to be 50 mmHg), and λ = 30 µm is the characteristic diffusion length. This model reflects diffusion‐limited oxygen delivery, in which oxygen tension drops rapidly with distance from the capillary and approaches critically low values at distances beyond ≈100 µm in metabolically active brain regions.^[^ ^42^, ^43^ ^]^

To simplify the concept, we can assume that tissue within 100 µm of a capillary is adequately oxygenated. Based on this, the fraction of hypoperfused cells (HypoR) in a spherical implant can be approximated as:$HypoR\;\left(r\right)={\left(\frac{r-100}{r}\right)}^{3}$(Equation 3), which indicates that as the radius (r) of the implanted cell mass increases, a greater proportion of cells are hypoperfused or hypoxic (Figure 3H). No hypoperfusion occurs when r ≤ 100 µm. At r = 100 µm, the volume of the sphere is ≈4.2 nL, which we define as the threshold volume (V_th_) for the onset of hypoperfusion (Figure 3I). We also calculated the implanted cell mass volume across a range of radii (Figure 3I, upper horizontal axis). For simplified interpretation in the context of cell transplantation experiments, we reformulated the HypoR function using total implanted cell volume instead of radius, $HypoR\left(v\right)=\frac{{\left(10\cdot {\left(\frac{3v}{4\pi }\right)}^{\frac{1}{3}}-1\right)}^{3}}{1000\cdot (\frac{3v}{4\pi })}$(Equation 2). This produces a skewed bell‐shaped curve (Figure S3, Supporting Information), where the intersection with the x‐axis represents V_th_, consistent with the volume threshold shown in Figure 3I (red dashed line).

To validate our mathematical modeling, we conducted experiments to examine how different injection volumes affect cell growth rates. These experiments were performed using a hyaluronic acid (HA) hydrogel matrix, which we previously demonstrated to support neural stem cell graft survival.^[^ ^44^ ^]^ C17.2 cells were injected at four distinct volumes into the hydrogel and cultured for 7 days followed by quantification of viable cells using bioluminescent imaging (Figure 3J). We observed that larger injection volumes resulted in more compact, spherical cell masses within the hydrogel, closely resembling in vivo transplantation conditions (Figure 3K). Analysis of the growth rate revealed significantly lower proliferation rates in the 0.5 µL (*p* = 0.0188), and 1.0 µL (*p* = 0.0343), groups compared to the 0.1 µL group, while no significant difference was observed between the 0.5 and 1.0 µL groups (Figure 3L and *p* = 0.9945). To test our hypoperfusion‐based mathematical model, we define the growth rate (G) as a function of the hypoperfused fraction (HypoR): *G*  =  *Gmax*  × · (1  −  HypoR)^3^, where Gmax is the maximal growth rate. As HypoR decreases, G approaches Gmax (Figure 3M). By substituting HypoR with the function derived from cell volume (as shown in Figure 3H), we expressed G as a function of injection volume (v, Figure 3M):$G=\mathrm{Gmax}\cdot (1-{)3{\left(1-\frac{100}{)3)3{\left(\frac{3v}{4\mathrm{pi}}\right)}^{3}}\right)}^{3})}^{3}$(Equation 3). Using the known *Gmax* value for C17.2 cells (Figure S4, Supporting Information), we fit the experimental data shown in Figure 3L to the theoretical curve. The resulting fit showed strong agreement, with an R‐squared value of 0.78 (Figure 3M). Fitting experimental data at different exponent values also confirms the current model provides the best fitting (Figure S5, Supporting Information). Thus, under our experimental setting, we could use Equation 2 or 3 to accurately predict the hypoperfused fraction or growth rate, respectively. These results demonstrate that injection volume significantly influences cell proliferation rate. Our hypoperfusion–volume model provides a useful tool for estimating hypoperfused fractions and predicting growth dynamics, offering practical guidance for optimizing cell transplantation protocols.

As cell concentration affects viability, we chose 2.5 × 10⁷ cells mL^−1^ for in vitro cell transplantation test, which is within the lower range of cell concentration used in relevant literature (Table 1, Figure S6A, Supporting Information). To obtain quantitative evaluation of the cell concentration's effect on viability/growth rate, we performed a concentration sweep (5 × 10⁶, 2.5 × 10⁷, 1.25 × 10^8^ cells mL^−1^, Figure S6A, Supporting Information) using our 100 µm ID injectors at a fixed volume (250 nL) into agarose gel (Figure S6B, Supporting Information, same as in Figure 3C,E) to decouple concentration from volume effects. Viability from day 1 through day 7 were quantified through BLI. We observed steady cell growth over time in the 5 × 10⁶ and 2.5 × 10⁷ cells mL^−1^ groups, but a sharp drop at Day 3 and stagnant growth in the subsequent days in the 1.25 × 10^8^ cells mL^−1^ group (Figure S6C, Supporting Information). Normalized plot reveals the significantly higher growth ratio in low cell concentration groups (Figure S6D, Supporting Information). This may be explained by the reduced viability due to crowding in a high cell concentration group. These data support the election of 2.5 × 10⁷ cells mL^−1^ for in vitro simulation and subsequent in vivo studies.

### 2.5 OPTRACE Enables the Development of Pulse‐Elevation for Intracortical Cell Injection

**Figure 4:**
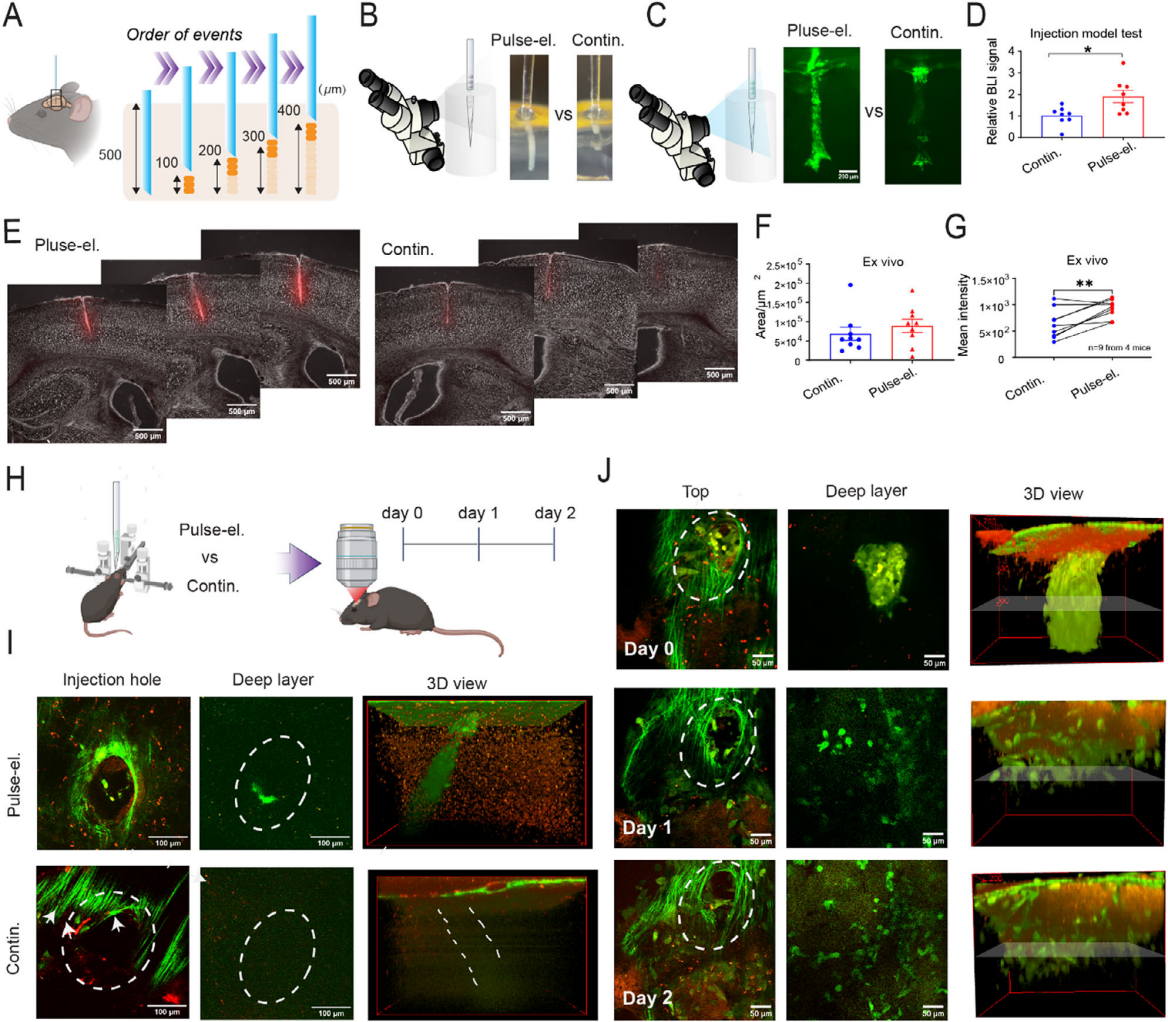
Pulse‐elevation model of injection improves cell retention. A) Schematic illustration of the pulse‐elevation strategy for transplanting cells into shallow depths of the brain. B,C) In vitro tests under bright field (B) or fluorescence (C) imaging through a stereoscope. Scale bar, 200 µm. D) Quantitative analysis of bioluminescence signal of C17.2 cells post‐injection (*p* = 0.0147, *n* = 8). Differences were analyzed by an unpaired T‐test. E) Representative coronal brain slices from Pulse‐elevation or Continuous injection groups. F) Quantification of needle track area in continuous and pulse‐elevation mode (*n* = 9 from 4 mice). G) Quantification of mean intensity in continuous and pulse‐elevation mode (*p* = 0.006, *n* = 9 from 4 mice). Differences were analyzed by paired T‐test. (*p < 0.05, **p < 0.01). H) The experimental design for in vivo TPFM. I) Comparison of the two methods of injection in terms of GFP‐positive C17.2 cells residing in needle track imaged right after transplantation under TPFM. Scale bar, 100 µm. J) Daily imaging the transplantation site for 3 days under TPFM.

To achieve OPTRACE for in vivo imaging, we intended to leverage intravital TPFM for visualizing donor cells during or after transplantation due to its advantages in spatial and temporal resolution for imaging scattering tissue.^[^ ^45^, ^46^, ^47^, ^48^, ^49^ ^]^ As the ideal imaging range of TPMF for mouse brain imaging is ≈450 micrometers,^[^ ^50^ ^]^ cells need to be implanted in shallow layers (L1‐L5) of the mouse cerebral cortex for them to be observable. However, as we learned from the above results, for intracortical transplantation of cells, retention rate will be a huge challenge due to the shallow depths (< 0.5 mm, Figure 3E). To address this issue, we hypothesize that pulsed cell injection with short intervals after each round of needle elevation during withdrawal of the needle will improve the retention rate due to the creation of space in needle track after each pulsed injection (**Figure** **4**A). We call this approach “pulse‐elevation” mode of cell injection.

We performed in vitro, *ex vivo*, and in vivo experiments to examine this hypothesis. As we learned that a smaller cell injection volume is preferred to keep the hypoperfused fraction (Figure 3H), we chose 100 nL as the total cell injection volume with a predicted hypoperfusion fraction based on Equation 2 (the predicted value, 0.278). Under the bright field (Figure 4B) or fluorescence imaging (Figure 4C), cells were closely monitored in the continuous or pulse‐elevation group, and we observed a significantly high number of cells that were retained in 0.6% agarose gel in the pulse‐elevation group (Figure 4D and *p* = 0.0147). Then we confirmed the efficacy of this approach by injecting cells into the mouse brain ex vivo (Figure 4E). Quantitative analysis of the needle track area showed no significant difference between the continuous and pulse‐elevation injection modes (Figure 4F and *p* = 0.7344). However, fluorescence signal intensity within the injection tract demonstrated that the mean intensity in the pulse‐elevation group was significantly higher than that in the continuous injection group (Figure 4G and *p* = 0.006). This indicates that while both injection modes cause similar levels of tissue damage, the pulse‐elevation mode enables greater cell retention within the injection tract. Moreover, the standard deviation of mean intensity in the pulse‐elevation group was notably lower (continuous mode = 279.9, pulse‐elevation mode = 170.7, Figure 4G), indicating reduced variability of injected cell number.

We then utilized TPFM to visualize the injection sites immediately after transplantation of C17.2 cells expressing GFP into the mouse cerebral cortex (Figure 4H). A substantial number of cells were retained in the needle track in the pulse‐elevation group compared to that in the continuous injection group (Figure 4I). These results indicate that the pulse‐elevation mode significantly improves the efficiency of cell delivery, holding promise for optimizing cell‐based therapies through minimizing cell loss and enhancing the precision of cell transplantation for therapeutic applications. Next, we extended the observation of implanted cells in the pulse‐elevation group through intravital TPFM. On Day 0, the implanted cell mass was visible (Figure 4J, row 1). On Days 1 and 2, a significant shift in cell distribution was observed (Figure 4J, row 2 and 3). While the needle track remained visible, transplanted cells were found in the surrounding tissue close to the injection site.

### 2.6 A Multicolor Cell Labeling Approach for OPTRACE at the Post‐Translational Phase

**Figure 5:**
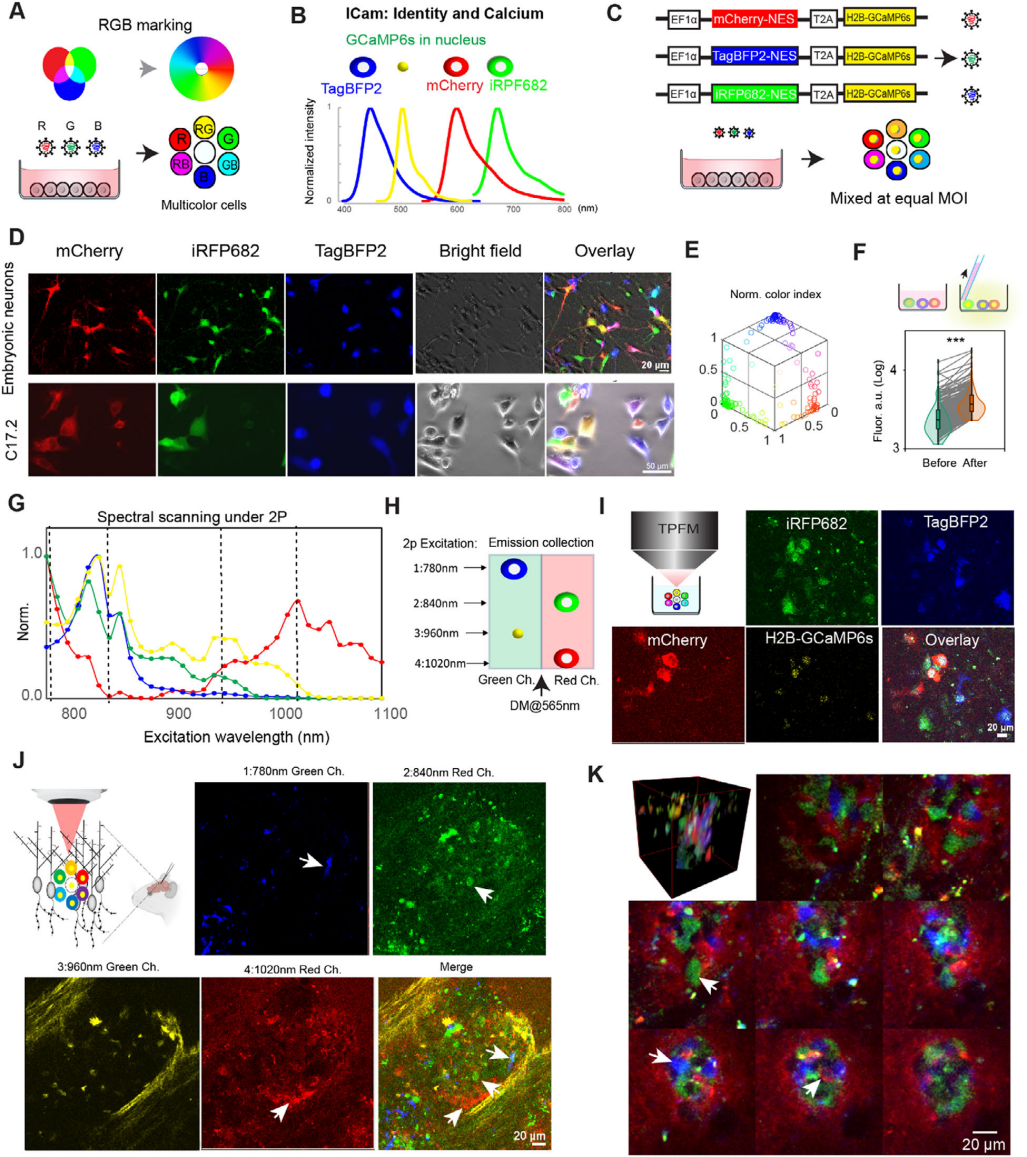
Tracking implanted cells at post‐transplantation phase through OPTRACE. A) The principle of RGB marking of cells. B) The component FPs in ICam and their well separated emission spectrum. IRFP682 and H2B‐GCaMP6s were false colored as green and yellow, respectively, for better visualization in merged multicolor mode. C) Lentiviral constructs for ICam and the principle of color mixing after cell transduction. D) Primary embryonic neurons from mouse cortex or C17.2 were transduced by ICam lentiviruses at equal MOIs, and the same field‐of‐view was imaged using different fluorescent filter sets to obtain individual and merged pictures. E) Normalized color index was mapped to 3D‐coordinate system for visualization of color distribution. Each dot represents one cell (*n* = 145 cells). F) Mechanical stimulation was applied by aspirating medium for induction of intracellular calcium rise. Fluorescence intensity of H2B‐GCaMP6s expressing cells were compared (*p* < 0.0001, *n* = 263 cells, Krustal‐ Wallis test). G) 2P excitation spectra for the four FPs used in ICam. H) Election of excitation wavelengths for each FP to unmix their signals. The location of the dot/cells indicates which channel is used for signal collection. DM: Dichroic mirror. I) ICam‐labeled hiPSC‐NPCs were imaged under TPFM using the setup in (H) for unmixing FP signals. J,K): ICam‐labeled cells (hiPSC‐NPC, J, or C17.2, K) were transplanted into mouse cerebral cortex. A representative of xy plane under TPFM showing the ICam labeled cells with separated color channels (arrows, J). Montage of the 3D stack of engraft site as a merge of red, green and blue channels showing ICam‐labeled C17.2 cells of different colors (arrows) at different depths under intravital TPFM.

For stem cell therapy, it is highly desirable to shed light into the black box of the post‐transplantation behavior of grafted cells from a methodological perspective. Taking advantage of TPFM for high‐resolution intravital brain imaging^[^ ^47^, ^51^ ^]^ and the availability of genetically encoded reporters, we previously developed an optical cell positioning system^[^ ^45^ ^]^ (oCPS) for intravital long‐term single cell tracking of endogenous neuroblasts, based on stochastic mixing of the three colors (Red‐Green‐Blue, RGB) of fluorescent proteins (FPs, **Figure** **5**A) working similarly to Brainbow.^[^ ^52^ ^]^ However, this strategy does not permit functional cell tracking. To solve this problem, we upgraded the RGB marking strategy to ICam (Identity and Calcium) at two aspects (Figure 5B): first, mVenus was replaced by an infrared FP, iRFP682^[^ ^53^ ^]^ which has a far‐red emission easily distinguishable from that of mCherry; second, we include a genetically encoded calcium indicator, GCaMP6s, targeted to the cell nucleus by fusing it to H2B. Thus, the four FPs (mCherry, TagBFP2, iRFP682, H2B‐GCaMP6s) used in ICam vectors are spatially and spectrally separated (Figure 5B). We used lentivectors encoding these FPs driven by a constitutive promoter (EF‐1alpha) (Figure 5C), which were packaged into lentiviruses (Figure S7, Supporting Information) for transduction of target cells (primary embryonic neurons or C17.2 cells, Figure 5D) at equal ratio to achieve maximal color variation,^[^ ^45^, ^54^ ^]^ resulting in the production of multicolor‐labeled cells with well‐scattered color hues (Figure 5E). We also confirm that all four FPs could be detected in transduced cells (embryonic mouse neurons or human iPSC‐derived neural progenitors, Figure S8A, Supporting Information). As for the performance of calcium indicator H2B‐GCaMP6s, we induced intracellular calcium rise by mechanical stimulation^[^ ^55^ ^]^ (pipetting up and down) and observed a significant rise of fluorescence intensity (*p* < 0.001, Figure 5F and Figure S8B, Supporting Information). This confirms the functionality of the calcium indicator in lentivirally transduced cells.

As OPTRACE relies on TPFM for cell tracking, we examine if these FPs could be detected and precisely unmixed under TPFM for intravital imaging. HEK293 cells labeled with individual ICam vectors were imaged under TPFM to obtain the two‐photon excitation spectra for each FP (Figure 5G). Based on their spectra, we chose for each FP the 2P wavelength that best separates it from others, namely, 780 nm for TagBFP; 840 nm for iRFP682; 960 nm for H2B‐GCaMP6s and 1020 nm for mCherry (Figure 5H). This setting enables precise unmixing of these four FPs in multiplex ICam labeled cells (Figure 5I and Figure S9, Supporting Information), confirming that we successfully established the labeling strategy that may allow functional single‐ cell tracking under TPFM. To obtain in vivo proof of the efficacy of our labeling strategy, we utilized the two‐photon wavelength setting depicted above to image ICam labeled cells (hiPSC‐NPC, Figure 5J or C17.2, Figure 5K) implanted into the mouse cerebral cortex using the pulse‐elevation approach. Numerous multicolor donor cells were observed at transplantation sites in either case (arrows). To further evaluate the application of ICam labeled cells for therapeutic use, we transplanted ICam‐labeled NSCs (C17.2) into the peri‐infarct region (within 0.5 mm to 1 mm from the ischemic core, Figure S10A,B, Supporting Information) 1 week after the induction of photothrombosis in the brain.^[^ ^6^, ^56^ ^]^ 2 days later, we observed long distance migration of implanted cells from transplantation sites toward the ischemic region (arrows in Figure 10C). High magnification observation of the graft reveals the presence of multicolor labeled NSCs with the three morphological color channels (mCherry, iRFP682 and TagBFP2) as well as the nuclear GCaMP6s signal (Figure S10D, Supporting Information). This result confirms the successful stable multicolor labeling of stem cells with ICam vectors that permits functional single‐cell tracking of therapeutic cells.

### 2.7 OPTRACE at the Post‐Translational Phase Reveals Active Interactions Between the Graft and Host Cells

**Figure 6:**
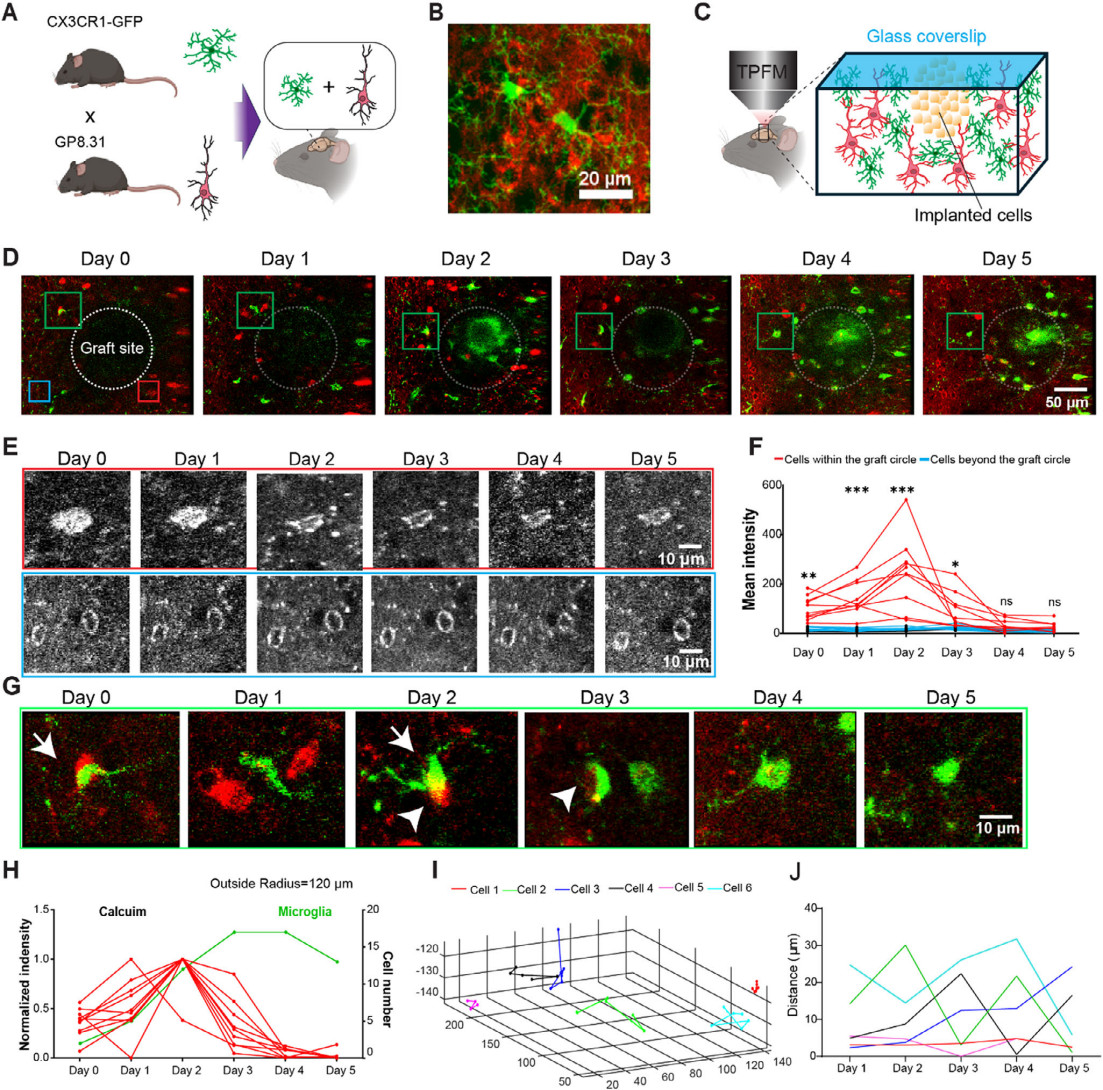
Temporal dynamics of calcium activity and microglial response following cell transplantaion. A) Schematic illustration of the generation of the double transgenic mouse line. B) Example morphology of neurons and microglia in the double transgenic mouse brain. C) Our experimental design: imaging graft‐host interactions under TPFM. D) Same area of the brain on different days post‐transplantation. Ddashed white circle shows the implantation site with a radius  of 120 µm Red tangles indicate regions adjacent to the graft site, whereas blue tangles represent distal regions. Scale bar, 50 µm. E) Magnified views of the same cells shown in (D) over time, showing calcium level in cells adjacent to (red box) and distal from (blue box) the graft site over 5 days. Scale bar, 10 µm. F) Quantification of calcium signal intensity in cells located within (red, *n* = 10 cells) and beyond (blue, *n* = 8 cells). day 0, *p* = 0.002, day 1 and day 2, *p* < 0.0001; day 3, *p* = 0.0105, day 4, *p* = 0.436, and day 5, *p* = 0.5425). Differences were analyzed by two‐way ANOVA followed by Tukey's test. (* *p <* 0.05, ** *p <* 0.01, **** *p <* 0.0001). G) An example microglia tracked over 5 days (highlighed in the green tangle shown in D). Scale bar, 10 µm. H) Dynamic change of microglia cell number (green) and its relationship to normalized calcium signal (red) within a 120 µm radius surrounding the graft over a 5‐day period. I) 3D spatial trajectories of six representative cells within the graft site. Each colored line represents the positional change of an individual cell over time, plotted in 3D space (x, y, z). Cell 2 is the cell shown in (G). J) Migration distance of individual microglial cells over a 5‐day period. Each colored line represents the tracked movement of a single microglial cell relative to its original position.

To investigate interactions between implanted cells and the host brain environment, we generated double‐transgenic mice by crossing CX3CR1‐GFP mice (labeling microglia with GFP) with the GP8.31 line (expressing the red‐shifted calcium indicator jReCO1a in neurons) (**Figure** **6**A). The resulting mice exhibited GFP‐labeled microglia and jReCO1a‐expressing neurons, as confirmed by histology (Figure 6B) and functional calcium imaging (Figure S11, Supporting Information). We then performed longitudinal imaging from Day 0 to Day 5 after implantation of mouse primary embryonic neurons in the cerebral cortex of this double transgenic mouse line CX3CR1: GP8.31 (Figure 6C). Host neurons (red) surrounding the graft site exhibited a sharp increase in fluorescence intensity shortly after transplantation, which gradually diminished over time (Figure 6D). This pattern was evident in longitudinally tracked neurons located near the graft site, defined by residing within the region of 120 µm radius centered on the implantation site, compared to those positioned beyong the circle (Figure 6E, upper and lower rows, respectively). Quantitative analysis (Figure 6F) showed that calcium signals in the peri‐graft region peaked on Day 1 or Day 2 and declined rapidly, returning to near‐baseline levels by Day 4. In contrast, neurons distal to the graft consistently displayed low calcium activity throughout the observation period, indicating that the elevated calcium signaling was spatially confined to the immediate graft vicinity. There are significant differences between these two groups on day 0 (*p* = 0.002), day 1 (*p* < 0.0001), day 2 (*p* < 0.0001), and day 3 (*p* = 0.0105). In parallel, microglia (green), the brain's first‐line responders to injury, were recruited to the graft area, as indicated by a progressive increase in their numbers within a circular region of 120 µm radius centered on the implantation site (Figure 6D,H). Microglial accumulation increased around Days 2–3, coinciding with the decline of calcium activity (Figure 6H; red line), and gradually decreased thereafter. (Figure 6H, green line) suggesting that early neuronal activity may contribute to initiating microglial recruitment. Comparable responses in calcium and microglia were observed in additional mice (Figure S12A–C,D–F, Supporting Information).

High‐resolution immunofluorescence imaging further revealed dynamic interactions between microglia and donor‐derived cells (Figure 6G). Microglial processes made direct contact with grafted cells and progressively engulfed cellular debris and damaged neurons (arrows, Figure 6G), consistent with active surveillance and phagocytic engagement in response to the graft. Longitudinal tracking of the same microglia enables us to plot their migration trace in 3D over time (Figure 6I), which displays high cell‐to‐cell variation in terms of their motility (Figure 6J). This may indicate different functional status requiring further investigation, potentially through molecular profiling techniques, such as scRNA seq.

## 3 Discussion

To overcome long‐standing barriers in stem cell transplantation—namely, imprecise delivery and poor visibility into post‐engraftment dynamics—we developed OPTRACE: a two‐step framework that integrates high‐precision, image‐guided injection with longitudinal single‐cell tracking in vivo. At the time of transplantation, OPTRACE enables real‐time visualization of cells through low‐cost, translucent glass pipettes under wide‐field or TPFM, allowing real‐time visualization and optimization of transplantation procedures. This is further enhanced by a novel pulse‐elevation injection technique, which improves engraftment in superficial cortical layers. Predictive modeling complements this precision approach by identifying injection parameters that maximize retention and minimize hypoxia stress. After transplantation, OPTRACE leverages multicolor genetic labeling and two‐photon imaging to monitor individual grafted cells as well as donor‐host interactions over time, revealing key biological processes including donor cell survival and migration, host microglial infiltration, and altered neuronal calcium dynamics at the graft‐host interface. By uniting these innovations, OPTRACE offers an accessible and modular platform to not only improve transplantation outcomes but also dissect the complex cellular interactions that underlie graft integration in the living brain.

OPTRACE establishes a new paradigm for intracerebral cell transplantation. Using wide‐field or TPFM to visualize fluorescently labeled cells during injection, OPTRACE enables real‐time, image‐guided delivery of cells with micrometer precision. Our custom apparatus (APP 2.0) – an open‐source pipette manipulator upgraded from our previous work^[^ ^29^ ^]^– allows fine control of pipette approach and pressure ejection while continuously imaging the brain (Figure 1A). This strategy draws on concepts from two‐photon–targeted patching (where dyes in the pipette render it visible under 2P)^[^ ^57^ ^]^ but extends them to cell transplantation. Notably, all hardware components (imaging scope, micromanipulator, pressure injector) are off‐the‐shelf or open‐source (e.g., APP 2.0), making this approach broadly accessible and adaptable to other laboratories. In short, OPTRACE combines widely available optics and actuators to produce a high‐precision transplantation platform, marking a clear advance over prior methods in stem‐cell grafting.

During the cell injection procedure, there are many factors that we did not evaluate in this study, such as geometry of injectors (ID, cone angle, taper profile, wall thickness or consistency of diameter of the glass pipettes) and material properties (rigidity, surface smoothness), which may affect cell survival. Smaller IDs leading to higher fluid shear stress that can damage cells, as evidenced by several studies on cell delivery.^[^ ^14^, ^58^, ^59^ ^]^ Our result is consistent with these prior studies, indicating that there is a threshold diameter (≈100 µm in our case) above which cell survival is not appreciably compromised. The cone angle (taper sharpness) of the pipette tip can also influence cell damage. A very steep, short taper (large cone angle) represents an abrupt geometric narrowing, which can create a sudden acceleration of fluid and high shear at the tip entrance.^[^ ^60^ ^]^ In our pipette fabrication, we did not vary the cone angle in a controlled way (all pipettes were pulled to have a fairly gradual taper of a few millimeters). A more systematic study (varying taper profiles, wall thickness, materials) could be valuable. Future work could employ simulations or machine learning on larger datasets to derive a multivariate model– for instance, varying diameter, taper angle, and injection speed to map out a response surface for cell survival.

Our system also integrates predictive mathematical modeling (retention–depth model and the hypoperfusion–volume model) to optimize transplantation parameters. We derived models relating cell retention to injection depth and hypoperfused tissue volume to injection volume. These models accurately predict outcomes: for example, deeper injections yield higher retention, while larger bolus volumes enlarge the zone of hypoxia. Quantitative simulation thus guides the choice of injection depth and rate that maximizes cell survival and minimize hypoperfusion area. This is important because in prior studies very few injected cells survive long‐term – often <5% retention after days^[^ ^9^ ^]^– and much cell loss is attributed to physical washout or vascular collapse. In regenerative medicine, such mathematical approaches have been advocated to refine therapy delivery: modeling cell–matrix interactions and perfusion can optimize dosing strategies in silico.^[^ ^61^ ^]^ Our results exemplify this synergy, showing that predictive models of retention and perfusion can be directly linked to improved engraftment. This approach complements preclinical studies optimizing intra‐arterial mesenchymal stem cells doses for stroke recovery.^[^ ^62^ ^]^ In practice, OPTRACE users can consult these models to optimize retention rate (Figure 3F) and improve viability of implanted cells (Figure 3M). Thus, the pairing of real‐time imaging with a quantitative model constitutes a powerful innovation for designing transplantation protocols. The current predictive models are validated in mouse/agarose conditions; applying it to other species requires re‐validation and possibly re‐parameterization. Future work might extend these models (e.g., to account for tissue compliance or repetitive injections, or to large animal cell transplantation scenarios) and integrate them into the OPTRACE software for on‐the‐fly planning.

A key enabler of OPTRACE is our multicolor and functional cell labeling strategy. By differentially labeling donor cells with combinations of fluorophores and calcium indicators, OPTRACE permits longitudinal functional single‐cell resolution tracking. For structural tracking, we previously developed the oCPS strategy^[^ ^45^ ^]^ which employ stochastic multicolor labeling, so that each transplanted cells can be uniquely identified by its color hue. Such spectral barcoding is well established in neuroscience: for example, Brainbow and its variants randomly colorize many cells, enabling reconstruction of individual cell morphologies and fates.^[^ ^63^ ^]^ To add a functional readout, we upgraded the oCPS by incorporating calcium indicators,^[^ ^64^ ^]^ creating the Identity and Calcium (ICam) constructs, which utilizes spectral and spatial multiplexing for precise identification of each FP. ICam also gives rich color diversity under 2P excitation. This multiplexed labeling dramatically increases throughput compared to monochromatic markers. Two‐photon calcium imaging then yields repeated measurements of each cell's activity. In this way, OPTRACE provides longitudinal structural and functional data: we can follow how specific grafted neurons survive, migrate, and become active (or silent) over time. Such capability contrasts sharply with traditional end‐point histology or static imaging. Two‐photon calcium imaging is a powerful approach to measure neuronal activity at high spatial resolution in neuroscience field^[^ ^47^, ^48^ ^]^ and our use of GCaMP within transplanted cells extends this to the grafted population. In short, OPTRACE's palette of labels (multicolor fluorophores plus functional indicator) combined with 2P microscopy offers an unprecedented window into the post‐engraftment life of single cells.

Using this platform, we uncovered dynamic graft–host interactions. For example, we observed marked changes in host neuron calcium dynamics after transplantation. Immediately adjacent to the graft site, many host neurons showed altered calcium transient frequency and amplitude compared to remote cortex, suggesting that the injection and presence of new cells modulate local network activity. These perturbations in neuronal calcium signaling may reflect tissue injury, synaptic remodeling, or inflammation, and they bear on functional integration. Similar neuroprotective effects from pretreated NSCs have been observed in stroke transplantation studies.^[^ ^65^ ^]^ Importantly, we also tracked the immune response: host microglia rapidly invaded the graft. In vivo two‐photon imaging revealed host‐derived microglial migrate into the transplant within days, consistent with literature.^[^ ^66^ ^]^ Through OPTRACE, we were able to track the migration of microglia, engulfing neurons with high calcium level and cleaning the debris (Figure 6). These observations suggest that microglia are actively sensing and responding to the new cells and any perturbations they induce. Furthermore, we found that neurons close to transplantation site displayed elevated calcium activity. Several in vivo two‐photon calcium imaging studies consistently demonstrate that neurons near sites of brain injury or implantation exhibit elevated calcium activity, while neurons farther away remain relatively unaffected. For example, Eles et al. (2018) showed that cortical neurons within ≈150 µm of an implanted electrode displayed acute calcium overload, unlike distant neurons.^[^ ^67^ ^]^ Similarly, Bibineyshvili et al. (2024) reported a localized calcium surge near a focal cortical injury with suppressed activity in remote regions.^[^ ^68^ ^]^ These findings align with our observation that neuronal calcium activity is heightened in the vicinity of the transplantation site, supporting the notion of spatially restricted excitability following cortical disruption. Overall, OPTRACE reveals a rich picture of graft integration: donor neurons gradually mature and form functional connections, while host microglia dynamically infiltrate, surveil, and possibly prune graft tissue. Such *live‐cell evidence* of neuronal and glial interplay could only be obtained with in vivo tracking; it provides mechanistic insight into why some grafts succeed or fail.

Compared to existing stem‐cell transplantation monitoring paradigms, OPTRACE offers major advantages in resolution, precision, and insight. Conventional approaches have primarily relied on post‐hoc histology or low‐resolution imaging. Clinical trials like MASTERS highlight the need for high‐resolution monitoring to track cell fate post‐delivery.^[^ ^69^ ^]^ For example, many studies including ours have used bioluminescent reporters (e.g., luciferase) to monitor graft survival noninvasively.^[^ ^44^, ^70^, ^71^ ^]^ While such methods can report whether transplanted cells are active on a whole‐animal level, they lack cellular detail. Likewise, magnetic resonance or PET imaging can track cell location macroscopically but cannot resolve individual cells or acute dynamics. TPFM has been used for observation after transplantation,^[^ ^66^, ^72^, ^73^, ^74^, ^75^, ^76^ ^]^ but the uniform cell labeling makes it hard to track highly dynamic cells over time. In contrast, OPTRACE provides high accuracy through multicolor cell labeling and couples imaging to the delivery itself. By seeing the pipette in real time, we dramatically reduce targeting errors: empirical recovery rates of transplanted cells are higher (e.g., 10–20% of cells remain versus <5% normally.^[^ ^9^ ^]^ Moreover, our spatial resolution is unparalleled: OPTRACE pinpoints cells to within tens of microns, and can track individual cells over time, something not possible with bulk imaging or single‐color labels. Temporally, OPTRACE is effectively continuous (images acquired many times per second during injection and repeatedly afterward), whereas modalities like bioluminescence integrate signals over minutes to hours. In summary, OPTRACE merges the cell‐level precision of microscopy with transplantation, giving both space‐ and time‐resolved control and readout.

While OPTRACE is currently demonstrated in preclinical mouse models, its principles hold translational potential for human brain research and regenerative therapies, such as stem cell transplantation in stroke or neurodegenerative disorders. A key limitation of TPFM is its effective imaging depth, typically restricted to ≈1 mm in cortical tissue due to light scattering and absorption.^[^ ^28^, ^77^ ^]^ To adapt OPTRACE for deeper human brain structures, the optical imaging component could leverage minimally invasive intraoperative multiphoton endomicroscopy, employing gradient‐index (GRIN) lenses or fiber‐optic probes, as demonstrated in our previous work for high‐throughput volumetric imaging in deep mouse brain regions.^[^ ^51^ ^]^ This approach has been demonstrated for in vivo intraoperative imaging of human brain tumor tissue during glioblastoma resection, as well as ex vivo on fresh samples from tumor and epilepsy resections, enabling subcellular‐resolution visualization of neoplasia and neural activity with low phototoxicity.^[^ ^78^, ^79^, ^80^, ^81^, ^82^ ^]^ Such techniques hold promise for real‐time guidance of cell delivery into peri‐infarct regions in stroke patients, potentially enhancing precision in clinical trials.^[^ ^83^, ^84^ ^]^ Furthermore, OPTRACE's predictive mathematical models for cell retention as a function of injection depth and hypoperfusion risk as a function of graft volume (Figures S1–S4, Supporting Information) are species‐agnostic and could inform human transplantations to minimize hypoxia and optimize engraftment, pending validation in larger animal models such as non‐human primates.^[^ ^85^ ^]^ These adaptations bridge preclinical optical innovations with clinical regenerative medicine, accelerating mechanistic insights and therapeutic development without overpromising immediate applicability.

In conclusion, OPTRACE represents a step‐change in how we perform and study neural cell transplantation. By integrating accessible imaging hardware and modeling, it achieves levels of precision and quantitative control previously unattainable. Our findings of graft‐host neuronal and immune dynamics open new avenues for optimizing cell therapies. Future work might extend OPTRACE to other cell types (e.g., oligodendrocyte progenitors or CAR‐T cells), explore optogenetic control in transplanted networks, or integrate magnetic resonance or ultrasound for even deeper guidance. Importantly, the open design (APP 2.0) invites the community to adapt OPTRACE widely, accelerating progress. OPTRACE is currently a preclinical research tool, with potential future translation pending further development. Ultimately, by pairing live optical readout with injection, OPTRACE not only improves engraftment outcomes, but also provides a rich experimental platform – a “living microscope” – for studying the biology of transplanted cells in situ.

## 4 Experimental Section

### Glass Pipette Preparation

Glass pipettes were fabricated from borosilicate capillaries (Drummond Scientific Wiretrol II / Plungers ‐ WIRETROL II, Cat#1‐5 UL ‐ 5‐000‐2005) using APPs. Briefly, glass capillaries were mounted in APPs with the glass axis colinear to the pull axis and centered in the coil hot zone, then secured by tightening the butterfly screw. A butane torch fitted with a vertical‐flame adapter was positioned beneath the capillary; the flame was ignited and adjusted to a stable, narrow plume until the glass softened and the capillary was drawn into two segments with sharp tapered ends. The pulled tips were allowed to cool briefly and were carefully harvested with forceps for downstream use (standard heat‐resistant PPE and flame safety procedures observed). Detailed schematics of APP 2.0 and a demonstration video on how to use APP 2.0 are provided in Figure S1 and Video S1, Supporting Information. Design files for APP 2.0 are available at https://github.com/liangy10/APP2. The pipette tips were subsequently beveled using a custom‐built rotary grinder adapted from an external hard drive motor.^[^ ^29^ ^]^ Pipettes were ultrasonically cleaned in 1 M NaOH (Sigma Aldrich, Cat#S5881) for 15 min and then rinsed twice in double‐distilled water, each for 15 min. Residual water inside the pipettes was removed by vacuum aspiration using a fine‐tip adapter. The dried pipettes were subsequently filled with mineral oil (Sigma Aldrich, Cat#M8410) using a backfilling technique and connected to a 10 µL Hamilton micro syringe (Hamilton, model 1701N) via a steel adapter. All pipettes were prepared on the day of use.

### Cell Cultures

HEK‐293 cells (RRID:CVCL_0063; obtained from ATCC in 2023) were cultured under identical conditions using DMEM supplemented with 10% fetal bovine serum (FBS, Sigma, Cat#F4135), 2 mM L‐glutmax (Gibco, Cat#35050‐061), and 1% penicillin–streptomycin (P/S; Sigma, P433). Mouse neural progenitor cells C17.2 (RRID:CVCL_4511) were sourced as previously described^[^ ^71^ ^]^ and maintained in high‐glucose Dulbecco's Modified Eagle Medium (DMEM,Gibco,Cat#11 965 118) supplemented with 10% FBS, 5% horse serum (Gibco, Cat#26050‐070), and 1% P/S at 37 °C in a humidified atmosphere containing 5% CO_2_. Primary cortical neurons were isolated from embryonic day 17.5 C57/BL6 mouse embryos, as previously described.^[^ ^86^ ^]^ Briefly, cortices were dissected and digested in 0.25% trypsin‐EDTA (ThermoFisher, Cat#25 200 056) containing 100 U mL^−1^ DNase I (Sigma Aldrich, Cat# 10 104 159 001) at 37 °C for 15 min, with gentle agitation every 5 min. Following enzymatic digestion, the tissue was gently triturated several times to dissociate aggregated tissue. After allowing the debris to settle for 2 min, the supernatant containing dissociated cells was transferred to a 15 mL centrifuge tube and centrifuged. The resulting cell pellet was resuspended in DMEM supplemented with 10% FBS and plated at a density of 1 × 10⁶ cells per well in 6‐well plates containing poly‐D‐lysine coated (50 µg mL^−1^, ThermoFisher, Cat# A3890401). Cells were initially cultured in DMEM with 10% FBS for 2 h to facilitate cell attachment. The medium was then replaced with Neurobasal medium (Thermo Scientific, Cat#21 103 049) supplemented with 2% B27 (Thermo Scientific, Cat#17 504 044). Neurons were maintained at 37 °C in a humidified incubator with 5% CO_2_. Human iPSC‐derived neural precursor cells (NPCs, obtained from Elixirgen Scientific in 2022, CIRM line CW50065, RRID: CVCL_DK86) were cultured following the Quick‐Neuron Precursor protocol (Elixirgen Scientific, Inc.). Cells were thawed, centrifuged, and plated onto 6‐well plates pre‐coated with Poly‐L‐Ornithine and laminin in NPC Medium (A). Cultures were maintained in NPC Medium under standard conditions (37 °C, 5% CO_2_) with media changes on days 1, 2, and 4. On day 7, cells were either passaged using a TrypLE or cryopreserved. All cultured cell lines were routinely tested to confirm mycoplasma‐free using PCR‐based assays.

### Cell Transduction by Lentivirus

All lentivectors were cloned and packaged by VectorBuilder and tittered by us. For multicolor lentiviral transduction, target cells were transduced with lentiviruses encoding distinct fluorescent proteins (e.g., iRFP‐682, mCherry, and BFP) at MOIs indicated in the results. The viral mix was supplemented with 5 µg mL^−1^ polybrene (VectorBuilder) to enhance transduction efficiency. After 24 h of incubation with the viral supernatant, the medium was replaced with fresh medium. Transduction efficiency was assessed by 72 h post‐infection using flow cytometry (BD LSR II) or florescence microscopy (Leica DMi8). For long‐term expression and lineage tracing, cells were sorted based on fluorescence profiles via fluorescence‐activated cell sorting (FACS, BD Aria II) expanded and maintained under standard conditions for downstream applications.

### Spectral Scanning Under Two‐Photon Imaging

HEK‐293 individually labeled with ICam lentivectors were imaged under two photons at excitation wavelength ranging from 780 to 1100 nm at step size 10 nm. Emissions were split by a dichroic mirror (565DCXR, Chroma) and detected by PMTs H16201P‐40//004, Hamamatsu) into green and red channel detected by PMTs. ROIs were drawn on the same area of images to measure intensity. After background subtraction, intensity was normalized to 0 to 1 based on minimal and maximum values.

### Cell Viability Test After Flowing Through Glass Pipettes

Following trypsinization with TrypLE (Gibco, Cat#12 604 021), cells were centrifuged at 1000 rpm for 5 min and resuspended at the target density in PBS (phosphate‐buffered saline, pH 7.4) (Gibco, Cat#10010‐023) supplemented with 1% bovine serum albumin (BSA, Sigma, Cat#A8412) and 0.5 mM EDTA (Ethylenediaminetetraacetic acid; Quality Biological, Cat#351‐027‐721). Cells were pipetted ten times per injection, with pipetted cells serving as the control group. For glass pipette diameter selection experiments, 2 µL of cell suspension (2.5 × 10⁷ cells mL^−1^) was injected into 18 µL of 0.4% Trypan Blue Solution (Thermo Fisher, Cat#15 250 061), gently mixed, and immediately analyzed using the Cellometer Auto 2000 Cell Viability Counter (Nexcelom Bioscience).

### Preparation of Brain Simulation Scaffold for Injection Depth and Volume Testing

For injection depth testing, cell suspensions (2.5 × 10⁷ cells mL^−1^) were injected at defined depths of 0.5 mm, 1.5 mm, and 3.0 mm into 0.6% low melting point agarose (Promega, Cat#v2111) using a glass pipette with a 100 µm tip diameter. A total volume of 1 µL was injected at a constant rate of 0.5 µL min^−1^, waiting for 2 mins before withdrawing. Following injection, cell viability within the agarose constructs was immediately assessed using bioluminescence imaging (BLI). For injection volume test, HyStem‐HP hydrogel (Sigma Aldrich, Cat#HYSHP020) was prepared according to the manufacturer's instructions for use in in vitro injection volume testing. To form the hydrogel, equal volumes of HyStem‐HP and Gelin‐S were mixed to obtain a 1:1 polymer blend. A total of 2.0 mL of this blend was then combined with 0.5 mL of 1× Extralink2 stock solution (resulting in a 4:1 polymer‐to‐crosslinker ratio, v/v), and the mixture was homogenized by gentle pipetting. Subsequently, 70 µL of the final pre‐gel solution was loaded into a custom gel holder fabricated from a trimmed 1 mL pipette tip: top inner diameter (ID): 2 mm; bottom ID : 5 mm; height: 9 mm. The filled holders were incubated at room temperature for 20–30 min to allow complete gelation. A series of defined volumes (0.1, 0.25, 0.5, and 1 µL) of cell suspension were injected into the hydrogel using a glass pipette with a 100 µm tip diameter. Injections were performed at a constant rate of 0.25 µL min^−1^ to a depth of 2.3 mm. Following injection, cells were transferred to 96‐well plate in incubator and cell viability within the hydrogel constructs was evaluated on 0, 3, and 7 days post‐injection using BLI. At each time point, D‐luciferin (Gold Biotechnology, eLUCK‐100; 15 µg mL^−1^ in 10 mM PBS, pH 7.4) was added directly to each well containing cells. Luminescent signals were acquired every 10 min using the Spark CYTO (Tecan). Peak luminescence values were subsequently used for quantitative analysis of cell survival.

Regarding the cell concentration optimization experiment, C17.2 cell expressing luciferase were prepared at 0.5, 2.5, and 12.5 × 10⁷ cells mL^−1^ and injected 250 nL into 0.6% low‐melting agarose and assessed by bioluminescence imaging (BLI) from Day 1 through Day 7 post injection. A 100 µm ID glass pipette was used for injection. Statistical comparisons followed our predefined plan (ANOVA/Kruskal–Wallis; boxplots: median/IQR with Tukey whiskers).

### Mathematical Modeling and Parameter Estimation

This work formalized two simple analytic models that relate (i) injection depth to cell retention, and (ii) injected graft volume to the hypoperfused fraction (and, by complement, normalized early graft growth). Each model has few interpretable parameters and is fit by nonlinear least squares to experimentally measured summaries. This work report goodness‐of‐fit (R^2^, MAE, RMSE), parameter uncertainty (bootstrap 95% CI), and residual diagnostics.

*Retention–depth model*. Rationale: Deeper insertions improve sealing and reduce reflux, increasing the fraction of cells retained at the target. Formulation: Let *d* be the insertion depth (mm) and *R(d)* the retained fraction (0–1). This work used a one‐parameter saturating function:

(1) $$R\left(d\right)=1-e^{-\beta d}$$

where β is a lumped parameter capturing tissue mechanics, needle tip/ID, cell suspension properties, and protocol (speed, pause). Data & preprocessing: For each depth, compute R as the site‐level retained fraction (retained cells / delivered cells) or a normalized imaging readout proportional to retained load. Exclude sites failing QC (e.g., leakage noted intra‐op). Estimation: β is estimated via nonlinear least squares (Matlab lsqcurvefit or Python scipy.optimize.curve_fit) with bounds [0, ∞). This work reported point estimate, 95% CI from 1000× nonparametric bootstrap (resampling sites), and fit metrics. Diagnostics: Plot residuals versus depth; test for trend (Spearman ρ); compute MAE and RMSE in retained fraction units. Interpretation & portability: β increases with improved sealing and/or lower reflux probability; for new tissues/species, β should be re‐estimated using the calibration assay.

*Hypoperfusion–volume model*. Rationale: Larger grafts increase diffusion distances and transiently outstrip local oxygen/vascular support, increasing the hypoperfused fraction and limiting early growth. Formulation: Let *v* be injected volume per site (µL). We model the hypoperfused fraction *HypoR(v)* with a flexible saturating rise:

(2) $$HypoR\left(v\right)=\frac{{\left(10\cdot {\left(\frac{3v}{4\pi }\right)}^{\frac{1}{3}}-1\right)}^{3}}{1000\cdot \left(\frac{3v}{4\pi }\right)}$$

If growth rate (G) was used as the as a function of the hypoperfused fraction (HypoR), this work supposed *G*  =  *Gmax*  × · (1  −  HypoR)^γ^, where Gmax is the maximal growth rate and Y is a sensitivity parameter (likely empirical, controlling how sharply G drops with increasing HypoR). As HypoR decreases, G approaches Gmax. By substituting HypoR with the function derived from cell volume (function 2), this work expressed G as a function of injection volume:

(3) $$G=\mathrm{Gmax}\cdot {\left(1-{\left(1-\frac{100}{{\left(\frac{3v}{4\mathrm{pi}}\right)}^{3}}\right)}^{3}\right)}^{\gamma }$$

Data & preprocessing: For each volume, compute either HypoR from imaging/markers (0–1) or G. If growth is measured, fit Equation (3); if hypoperfusion is measured, fit Equation (2). Estimation & model selection: Fit (v, γ) by bounded nonlinear least squares (v > 0, γ > 0). Compare candidate exponents (e.g., fixed γ = 1,2,3) and the free‐γ model using R^2^, RMSE, and residual structure; keep the simplest model whose residuals show no volume‐dependence. Report parameter CIs via bootstrap. Diagnostics: Residuals versus volume; lack‐of‐fit test; sensitivity sweep ≈±20% of each parameter to visualize robustness. Interpretation & portability: v decreases with poorer local perfusion or higher cell oxygen demand; γ > 1 suggests supra‐linear growth of diffusion limitation with size. Re‐estimate γ when cell type, matrix, or vascularity change.

### Pulse‐Elevation Mode Injection Into Agarose Gel And Quantification

C17.2‐luc2⁺ cells (2.5 × 10⁷ cells mL^−1^) were injected into 0.8% low‐melting‐point agarose. The target depth for the cell transplantation was set at 500 µm. Before injection, glass pipette was extended beyond the target depth to 1000 µm and then retracted back to a target depth of 500 µm. For the pulse‐elevation mode, a five‐step infusion protocol was employed to deliver a total volume of 100 nL at a constant rate of 0.1 µL min^−1^. A total of 20 nL was injected at sequential depths of 0.5, 0.4, 0.3, 0.2, and 0.1 mm, with a 20‐s pause between steps. During each injection, the glass pipette was gradually retracted, and after the final step at 0.1 mm, it was held in place for 5 min to promote diffusion and reduce backflow. In the continuous mode, the same cell suspension, depth, injection rate (0.1 µL min^−1^), and total volume (100 nL) were used, but the entire volume was delivered in a single uninterrupted step. Retained cells were quantified using bioluminescence imaging (BLI).

### Comparison of Injection Methods into Dissected Mouse Brain and Analysis

HEK‐293 cells (3.0 × 10⁷ cells mL^−1^) were labeled with 25 µM Vybrant DiI cell‐labeling solution (Invitrogen, Cat#V22889) following the manufacturer's protocol. Briefly, 5 mL of 25 µM DiI solution was added to the cell pellet after enzymatic digestion and centrifugation. The cell suspension was gently mixed by pipetting and incubated at 37 °C for 20 min. After incubation, cells were centrifuged and washed three times with sterile PBS to remove excess dye. The labeled cells were then resuspended to a final concentration of 2.5 × 10⁷ cells mL^−1^. A total volume of 100 nL of DiI‐labeled HEK‐293 cell suspension was injected into predefined brain regions (ML: ±1.5 mm; AP: ±1.2 mm and ±2.5 mm; DV: –0.5 mm) at a constant rate of 0.1 µL min^−1^. Injections were performed using two modes: pulse‐elevation mode for the right hemisphere and continuous mode for the left hemisphere. Immediately following the injection, brains were fixed in 4% paraformaldehyde and cryoprotected in 30% sucrose in PBS. Coronal brain sections (30 µm thickness) were prepared using a cryostat (CryoStar NX50, Epredia) and imaged using a fluorescence microscope (Leica DMi8) to evaluate the distribution of injected cells.

For ex vivo analysis, 25 µm‐thick brain slices containing DiI‐labeled transplanted cells were imaged using a Leica fluorescence microscope. All images were acquired under identical settings, including exposure time and gain, to ensure consistency across samples. The images were imported into ImageJ (Fiji), and brightness and contrast were adjusted uniformly using the “Brightness/Contrast” tool. To quantify the injection site, the DiI‐labeled injection track was manually outlined using the “Freehand Selection” tool, and the selected region of interest (ROI) was added to the ROI Manager. The area and mean fluorescence intensity within each injection track were measured and exported for further analysis.

### Mice

All experimental protocols at the University of Maryland, Baltimore, were conducted according to the National Institutes of Health guidelines for animal research and approved by the Institutional Animal Care and Use Committee at the University of Maryland, Baltimore. Mice were group housed with littermates until craniotomy surgery, after which they were singly housed. Mice were maintained on a 12–12‐h (6 a.m.–6 p.m.) light–dark cycle.

### Cell Transplantation and Cranial Window Installation

Male C57BL/6J mice or double transgenic mice breed from CX3CR1‐GFP (JAX mice strain # 0 05582) and GP8.31(JAX mice strain #03 0526) were anesthetized with isoflurane (5% for induction and 1.5% for maintenance) and positioned in a stereotaxic frame (RWD Stereotaxic Instruments). A circular craniotomy (3 mm diameter) was performed over the primary visual cortex (V1), centered 2.5 mm lateral and 0.5 mm anterior to the Lambda suture. A 100 nL volume of the cell suspension (2.5 × 10⁷ cells mL^−1^) was injected into the cortex at coordinates: AP = −2.5 mm, ML = 2.5 mm, DV = 0.5 mm using a 100 µm ID glass pipette at a rate of 0.1 µL min^−1^. After injection, a 3‐mm diameter circular coverslip adhered to a 3.5‐mm diameter donut‐shaped coverslip (Assistent Deckgläser Microscope Cover Glasses) was affixed over the craniotomy using black dental cement (Contemporary Ortho‐Jet). A custom titanium headpost was then secured to the skull with dental cement. Mice were allowed to recover on a heating pad before being returned to their home cages.

### Two‐Photon Imaging and Analysis

Mice were kept on a warm blanket (37 °C) and anesthetized using 0.5% isoflurane and Imaging was performed with a custom‐built two‐photon microscope with a resonant scanner. Each experimental session lasted 45 min to 2 h. Multiple sections (imaging planes) may be imaged within the same mouse. Fluorophores were excited by a different wavelength with a femtosecond laser system (Chameleon Discovery, Coherent) that was focused by an Olympus ×25, 1.05‐NA objective. Emitted fluorescence photons reflected off a dichroic longpass beam splitter (FF705‐Di01–25 × 36, Semrock) and were split with a dichroic mirror (565DCXR, Chroma) and detected by PMTs H16201P‐40//004, Hamamatsu) after filtering with a 510/84‐nm filter (84‐097, Edmund) for the green channel and two 750SP filters (64‐332, Edmund) for the red channel. Images were acquired using ScanImage^[^ ^63^ ^]^ (Vidrio Technologies). 3D z‐stack images were acquired at a step size of 2 µm along the z‐axis. After imaging, animals were returned to their home cages for recovery. For longitudinal imaging at subsequent time points, the same imaging parameters and field of view were used for data acquisition from the same animal. In mice transplanted with multicolor‐labeled cells, wavelength optimization was first performed to determine the most effective excitation conditions for each fluorophore. Based on the results, specific wavelengths were used for each channel: 780 nm for BFP, 842 nm for iRFP‐682, and 1020 nm for mCherry and GcaMP6s. The laser power ranges from 4 to 16.8 mW.

For real‐time imaging cell injection process into the dissected mouse brain, C17.2 cells labeled with GFP (2.5 × 10^7^ mL^−1^) were injected through glass pipette (ID = 110 µm) under two‐photon imaging with excitation at 960 nm. The dissected mouse brain was immersed in cold artificial cerebrospinal fluid CSF (aCSF) on ice. ACSF was prepared by dissolving 124 mM NaCl, 3 mM KCl, 1.25 mM NaH_2_PO_4_·H_2_O, 1 mM MgSO_4_·7H_2_O, 2 mM CaCl_2_·2H_2_O, 26 mM NaHCO_3_, and 10 mM D‐glucose in distilled water. After adjusting the final volume to 1 L with distilled water, the aCSF was used immediately or stored at 4 °C for short‐term use.)

Imaging data were processed with custom programs written in MATLAB (Mathworks) and Fiji.^[^ ^87^ ^]^ Raw two‐photon imaging data were imported into ImageJ for the generation of composite images. 3D image reconstructions were obtained using the “3D Viewer.” Fluorescence intensity of calcium signals in local neurons was quantified by drawing ROIs over the soma of neurons and the background was subtracted. For microglial tracking, first, we identified the same cell across time points using consistent local anatomical landmarks and stable reference cells, including non‐activated local neurons and stationary neighboring cells. Then we mapped their location into one coordinate system. The x, y, and z coordinates of the same microglial cell were recorded across time within the same field of view. Spatial migration and migration distance of microglia were then analyzed using custom‐written scripts in MATLAB.

### Statistical Analysis

Data analysis was performed using a combination of standard functions and custom scripts in MATLAB, Prism 10 (GraphPad Software Inc. US) or PAST.^[^ ^88^ ^]^ The data were tested for normal distribution using the Shapiro–Wilk test. Parametric tests were used for normally distributed data, and non‐parametric tests were applied to all other data. Bar graphs and mean ± SEM were used to describe the data with normal distribution, while boxplots and median ± IQR were used to describe the non‐normally distributed data. Boxplots represent median and 25th ‐ 75th percentiles, and their whiskers are shown in Tukey style (plus or minus 1.5 times IQR). For comparisons among multiple groups under a single factor, if the data were normally distributed, one‐way ANOVA followed by Turkey's test was performed. If data were not normal distributed, a nonparametric test (Wilcoxon signed rank test) was used to examine paired data (Figure 4F). The statistical significance was defined as ∗p < 0.05, ∗∗p < 0.01, ∗∗∗p < 0.001, ****p < 0.0001, respectively. Medians, IQR, means and SEM are reported throughout the text.

## Conflict of Interest

The authors declare no conflict of interest.

## Author Contributions

Y.L. conceived the idea and designed the research. J.W. performed cell culture, ex vivo, transplantation, and in vivo imaging experiments as well as data analysis. H.T. contributed in vitro test, in vivo imaging and analysis. C.R., M.W., and D.G. designed and tested the upgraded APP. G.Q, C.C, M.J., and P.W. contributed to design and imaging of animal experiments. X.F. contributed to flow cytometry. Y.L. and P.W. supervised research. Y.L. and J.W. wrote the manuscript.

## Materials & Correspondence

Design files: design files are available here: https://github.com/liangy10/APP2. Codes for modeling are available in Supplemental information. All data are available from the Lead Contact, Yajie Liang (Yajie.liang@som.umaryland.edu), upon request.

## Supporting information



Supporting Information

Supplemental Video 1

Supplemental Video 2

## Data Availability

The data that support the findings of this study are available from the corresponding author upon reasonable request.
