# Supplementary material for: OPTRACE: Optical Imaging–Guided Transplantation and Tracking of Cells in the Mouse Brain
Source: Adv Sci (Weinh). 2025 Dec 12;13(12):e14183. doi: 10.1002/advs.202514183 (PMC12948288; doi:10.1002/advs.202514183)
Supplement: Supplementary file 1 — Supporting Information [file ADVS-13-e14183-s003.docx]

**OPTRACE: Optical Imaging–Guided Transplantation and Tracking of Cells in the Mouse Brain**

Jinghui Wang^1^, Honglin Tan^1^, Colleen Russell^1^, Mikolaj Walczak^1^, Dawei Gao^1^, Guanda Qiao^1^, Xiaoxuan Fan^2^, Chengyan Chu^1^, Miroslaw Janowski^1^, Piotr Walczak^1^ and Yajie Liang^1,^*

1. Department of Diagnostic Radiology and Nuclear Medicine, University of Maryland School of Medicine, Baltimore, Maryland 21201, United States

2. Department of Microbiology and Immunology, University of Maryland School of Medicine, Baltimore, Maryland 21201, United States

*Corresponding author: [Yajie.liang@som.umaryland.edu](mailto:Yajie.liang@som.umaryland.edu)

**Supplementary Figure 1-12**

**Supplementary Table 1.**

**Supplementary codes for modeling.**


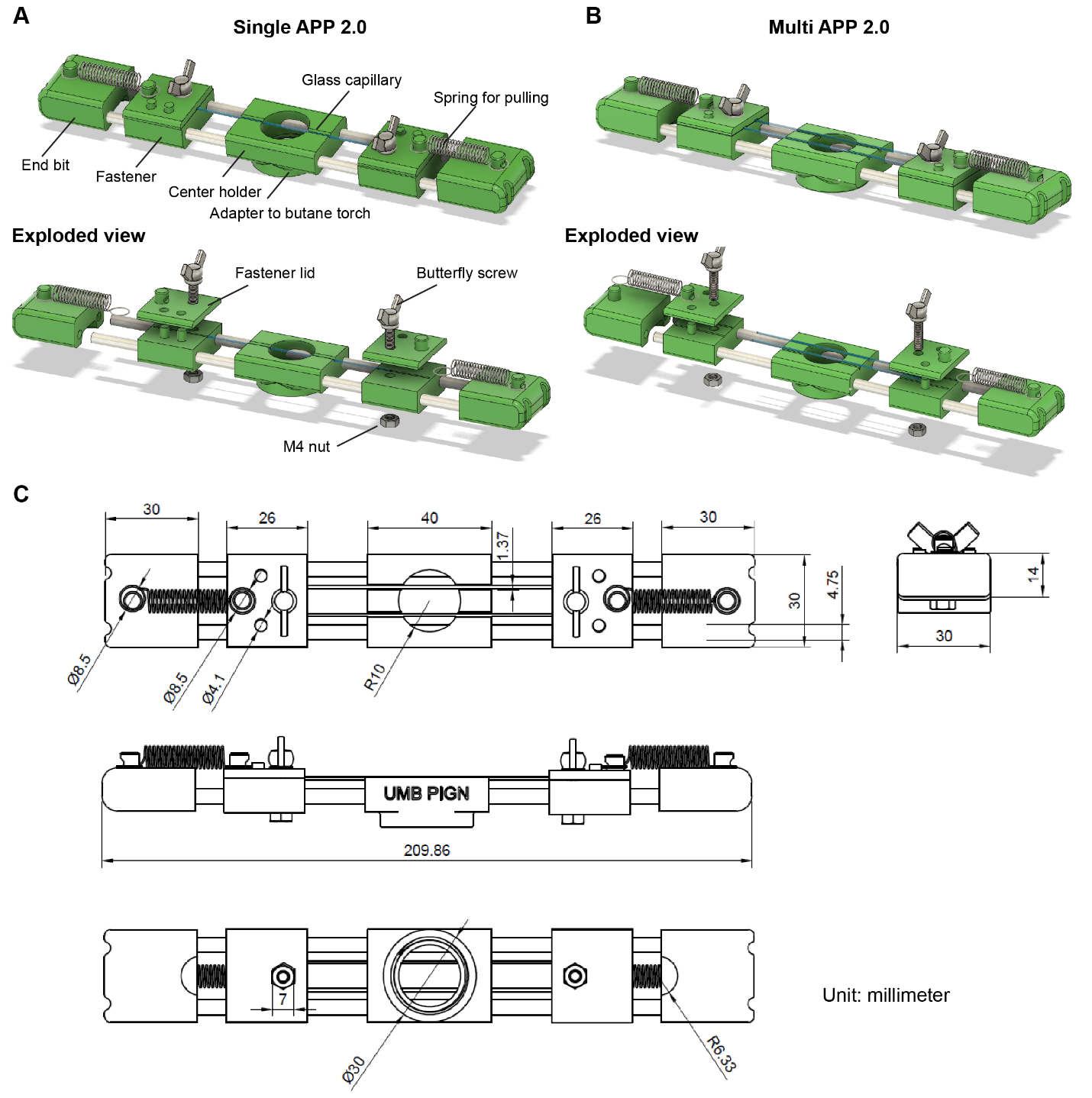


**Supplementary Figure 1. Detailed description of APP2.0. A.** Single channel APP 2.0 design and exploded view. B. Multi-channel APP 2.0 design and exploded view. **C**. Dimensions of APP2.0 drawing at different view angles.

**
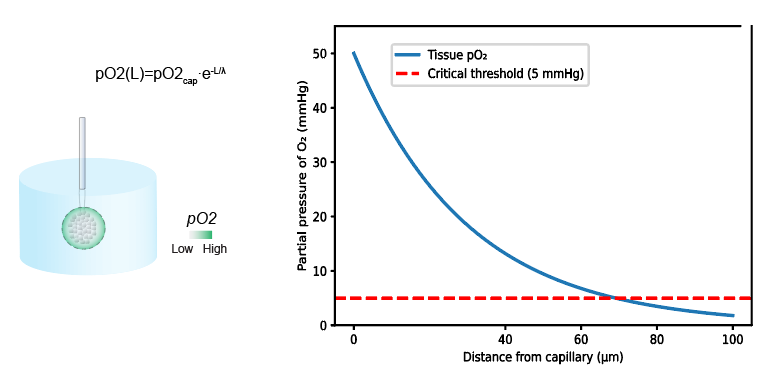
**

**Supplementary Figure 2. The mono-exponential decay model for evaluating oxygen diffusion in tissue.** L is the distance from the nearest capillary, pO₂cap is the initial oxygen partial pressure near the capillary (typically assumed to be 50 mmHg), and λ = 30 µm is the characteristic diffusion length. This model reflects diffusion-limited oxygen delivery, in which oxygen tension drops rapidly with distance from the capillary and approaches critically low values at distances beyond ~100 µm.

**
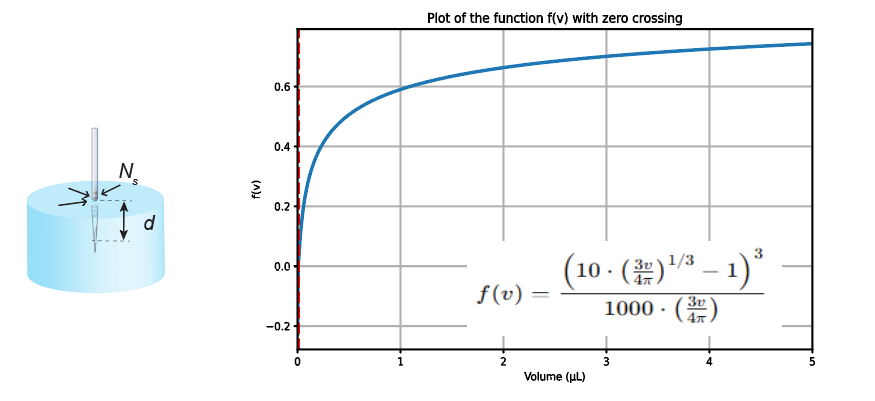
**

**Supplementary Figure 3.** **HypoR as a function of implanted cell volume.** The intersection with the x-axis represents Vₜₕ.


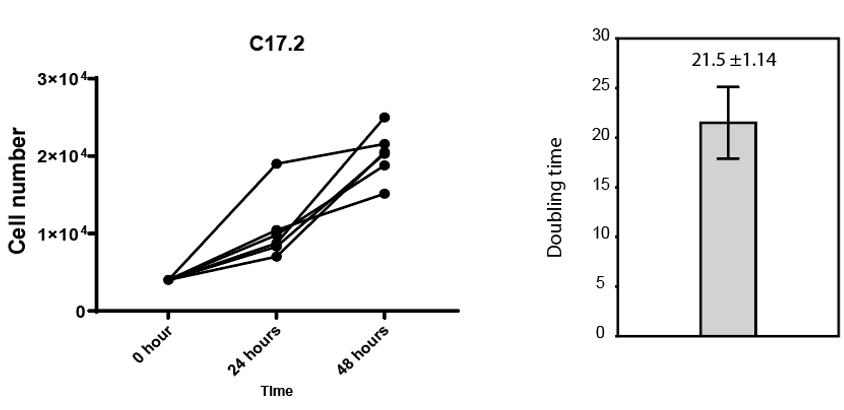


**Supplementary Figure 4. Determination of Gmax value for C17.2.** Cells were cultured in tissue culture plate and cell number was quantified by cck8 at different timepoints. Doubling time was calculated by Time x Log(2)/log(Nt/N0), in which Nt as cell number at Time t and N0 as starting cell number.


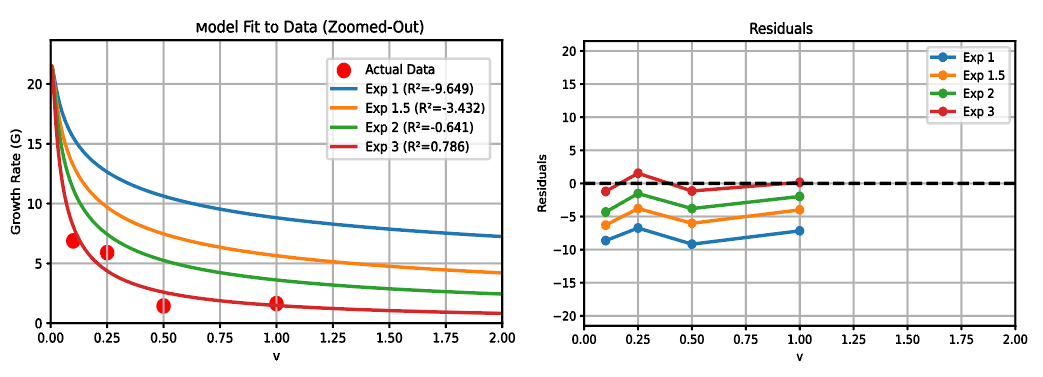


**Supplementary Figure 5. Fitting experimental data to formula with different exponential** γ**.** Exponential (γ) at 3 gives best fitting evidenced by largest R square value (left panel) and least residual (right panel)


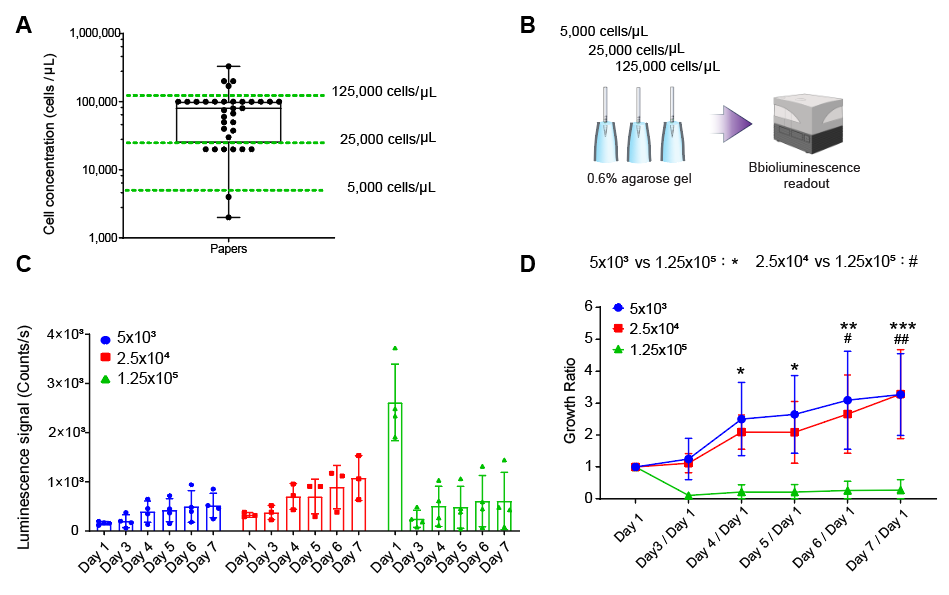


**Supplementary Figure 6. The effect of cell concentration on viability of transplanted cells over time. A.** Survey of reported cell injection concentrations in the literature showing a wide range from 10³ to 10⁶ cells μL⁻¹, with the experimental concentrations (5 × 10³, 2.5 × 10⁴, and 1.25 × 10⁵ cells μL⁻¹) highlighted in dashed green lines. **B.** Schematic illustration of the experimental setup, where FCIV-Luc2⁺ C17.2 cells at three concentrations were injected into 0.6% low-melting agarose and assessed by bioluminescence imaging (BLI). **C.** Luminescence intensity (counts s⁻¹) over 7 days showing that the high-concentration group (1.25 × 10⁵ cells μL⁻¹) initially exhibited stronger signals but declined sharply by Day 3, whereas the low- (5 × 10³) and medium-concentration (2.5 × 10⁴) groups maintained stable or gradually increasing signals. **D.** Growth ratio (Day n / Day 1) analysis indicating significantly faster proliferation in the low- and medium-concentration groups compared with the high-concentration group, particularly after Day 6 and Day 7, respectively. Data are presented as mean ± SD (n = 4, 3, 4). Statistical significance: ** p < 0.05, ** p < 0.01, *** p < 0.001* for 5 × 10³ vs 1.25 × 10⁵ cells μL⁻¹; *# p < 0.05, ## p < 0.01* for 2.5 × 10⁴ vs 1.25 × 10⁵ cells μL⁻¹ (two-way ANOVA with Tukey’s multiple comparisons test). When analyzed by one-way ANOVA for individual time points (Day 3–Day 5), the following p-values were obtained: Day 3 / Day 1: p = 0.0136 (5 × 10³ vs 1.25 × 10⁵), p = 0.0359 (2.5 × 10⁴ vs 1.25 × 10⁵); Day 4 / Day 1: p = 0.0072 (5 × 10³ vs 1.25 × 10⁵), p = 0.0295 (2.5 × 10⁴ vs 1.25 × 10⁵); Day 5 / Day 1: p = 0.0123 (5 × 10³ vs 1.25 × 10⁵), p = 0.0601 (2.5 × 10⁴ vs 1.25 × 10⁵).


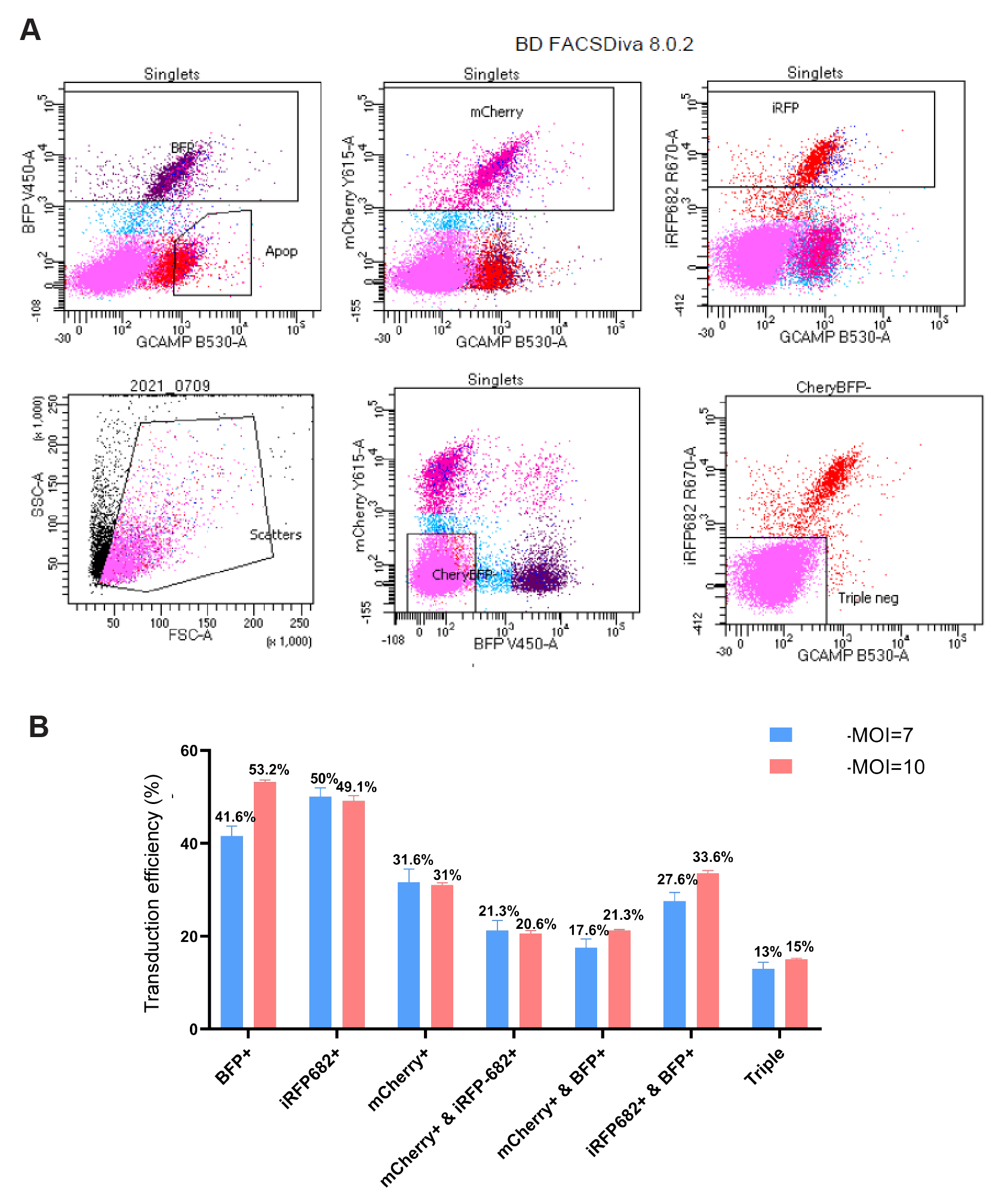
**Supplementary Figure 7. Characterization of ICam lentivectors for mixed transduction of HEK293 cells. A**. All four FPs in ICam vectors were readily detected through flow cytometry. **B**. Quantification of transduction efficiency for different populations of cells labeled with single, double or triple colors in transduced HEK293 cells. MOI: Multiplicity of Infection.


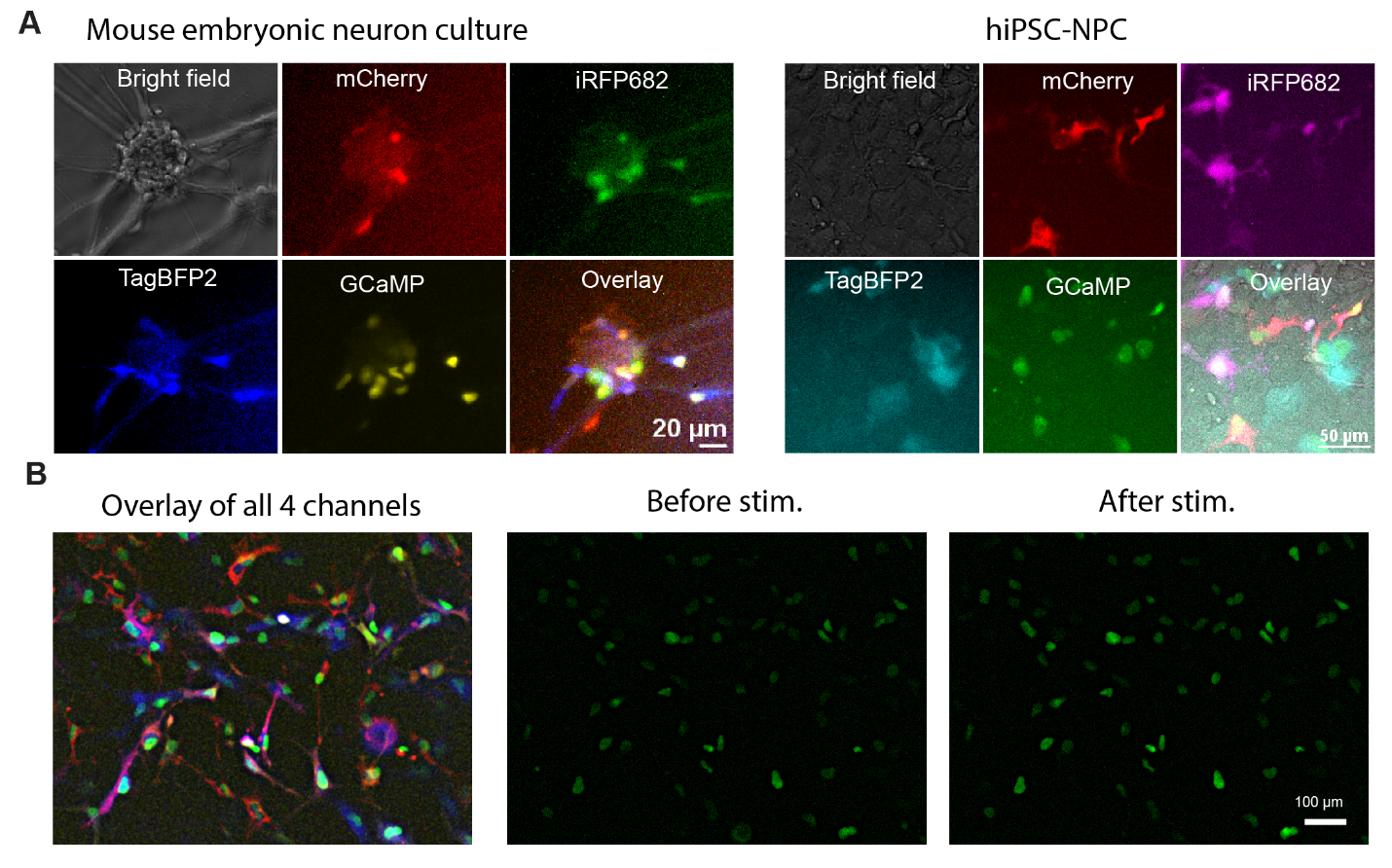


**Supplementary Figure 8. Confirmation of FPs and calcium indicator functionality.** **A**. All four FPs in ICam vectors were readily detected in transduced cells (embryonic mouse neurons or human iPSC-derived neural progenitors, NPC). **B**. In ICam transduced iPSC-NPC, mechanical stimulation induced the rise of green fluorescence (Right two panels).


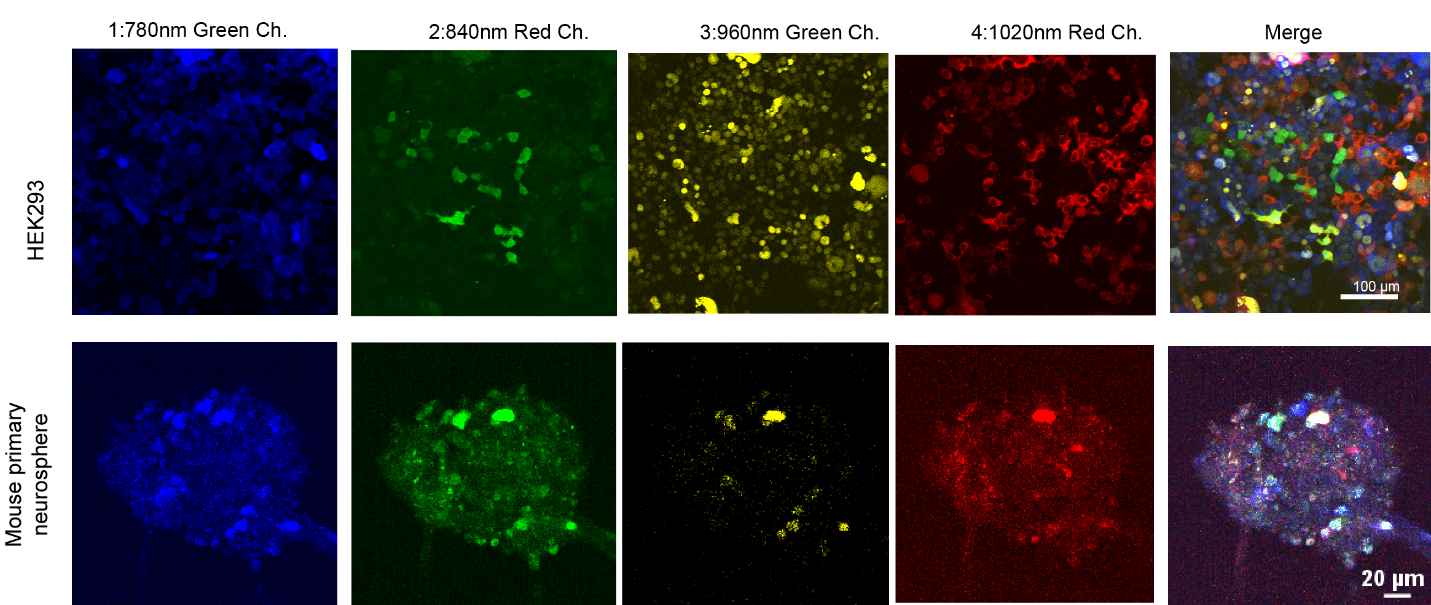


**Supplementary Figure 9. 2P acquisition settings.** Unmixed FP signals were extracted using our special excitation wavelength set in multiplex ICam labeled HEK293 or mouse primary neurospheres.


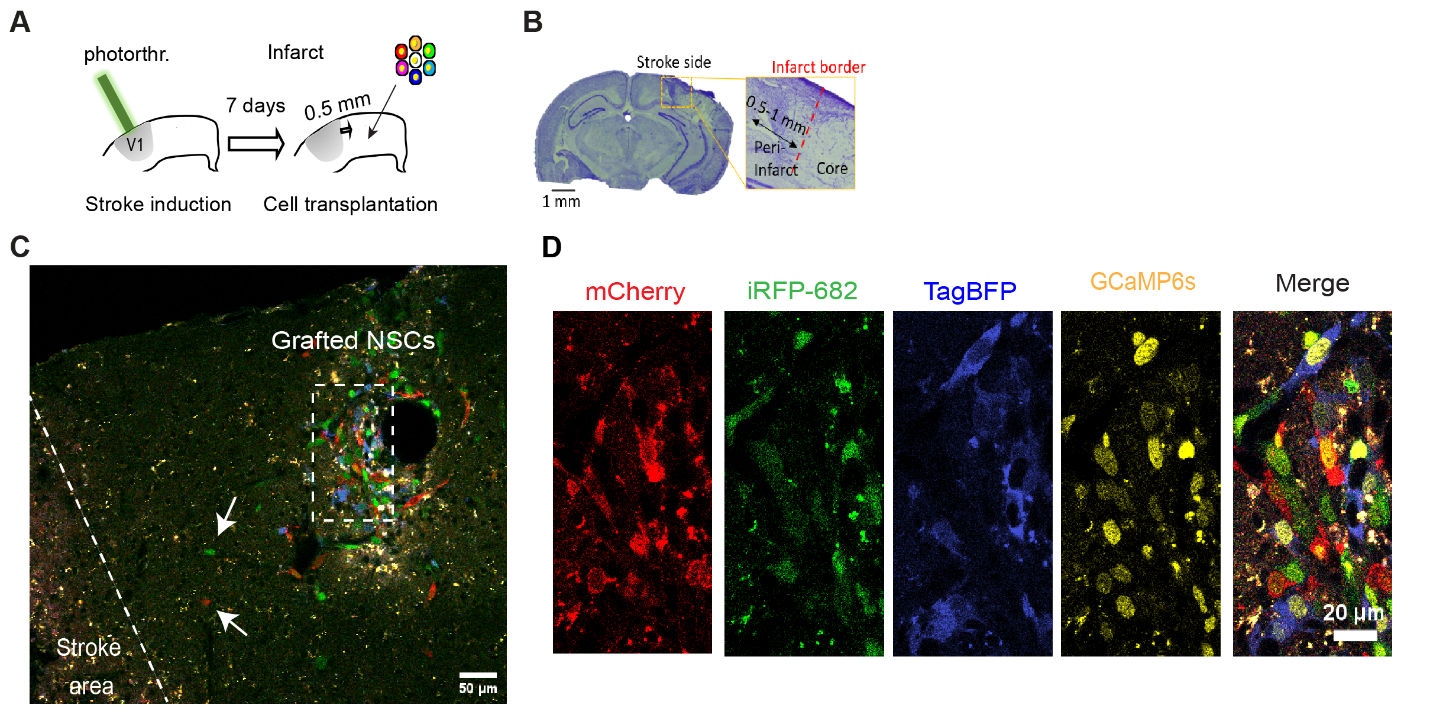


**Supplementary Figure 10. Using ICam labeled cells for therapy of a stroke model.** A, B. Photothrombosis was induced (A) one week before the transplantation of ICam-labeled C17.2 cells into peri-infarct region (B). C. Migration of ICam-labeled NSCs (arrows) toward ischemic region two days after transplantation. D. Zoom in of dashed region in (C) with the breakdown of color channels.


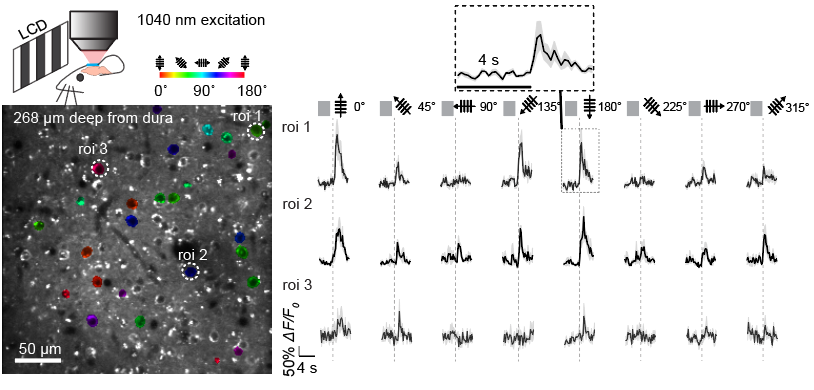


**Supplementary Figure 11. Calcium responses from neurons expressing jRGECO1a.** We imaged Layer 2/3 cortical neurons in the mouse primary visual cortex with 1040 nm 2-photon laser while the animal was exposed to drifting grating visual stimulation 3 weeks after cranial window installation. Cortical neurons in V1 show clear orientation selectivity (left panel) and there are big-magnitude responses from these neurons that could be as high as 150 ΔF/F% above base line (right panel).


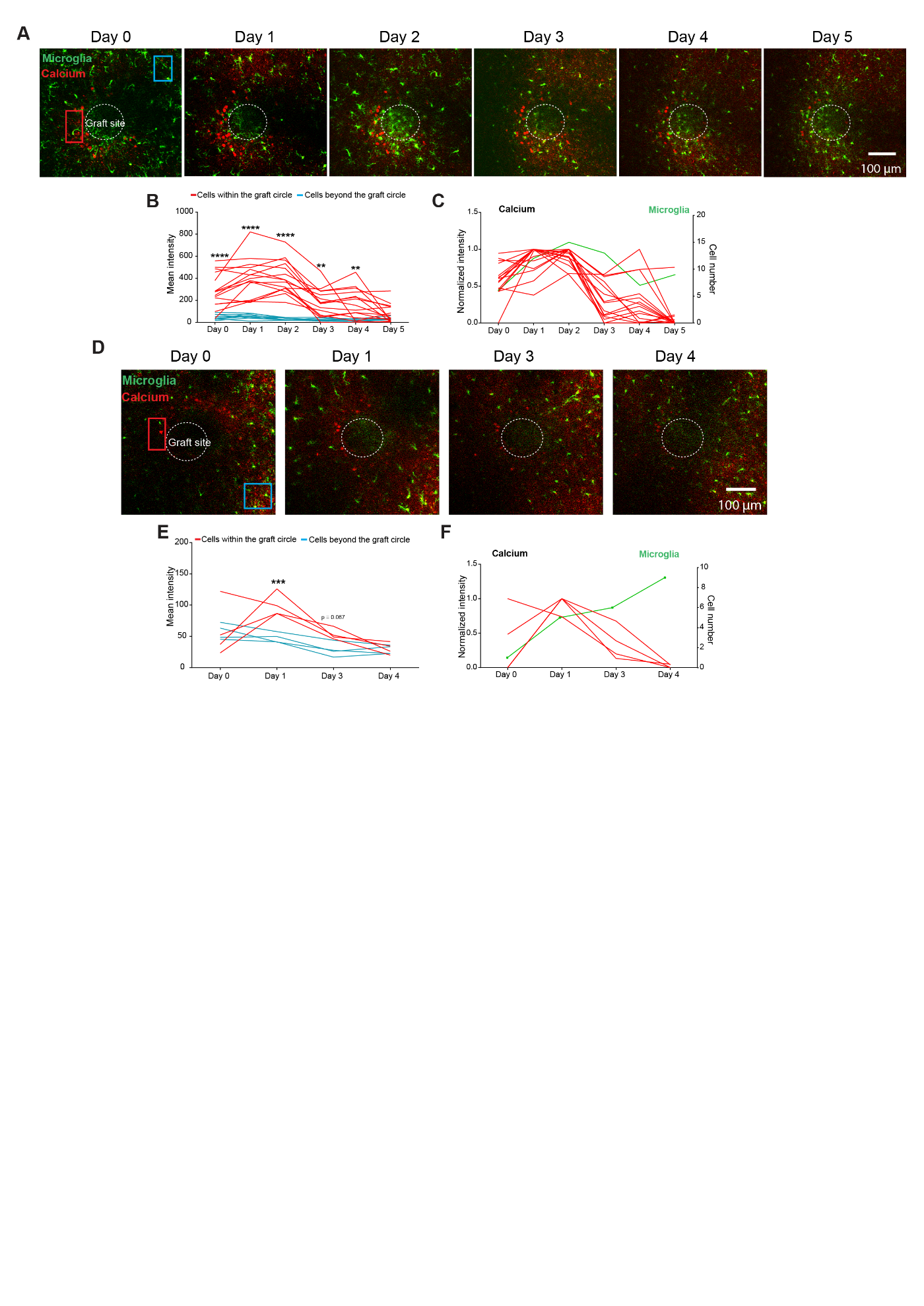


**Supplementary Figure 12. Temporal dynamics of calcium activity and microglial response following cell transplantation.** (A-C) from one mouse, and (D-F) from another mouse. (A, D) Representative two-photon images of the same brain regions across different days post-transplantation. The dashed white circle indicates the graft site (radius: 125 µm). Red and blue boxes mark representative regions within and beyond the graft-adjacent zone, respectively. Scale bars, 100 µm. (B, E) Quantification of calcium signal intensity in cells located within (red) and beyond (blue) the graft-adjacent region. For panel B (n = 16 cells within, n = 10 cells beyond): day 0–2, p < 0.0001; day 3, p = 0.0010; day 4, p = 0.0019; day 5, p = 0.3353. For panel E (n = 5 cells each): day 0, p = 0.5836; day 1, p = 0.0002; day 3, p = 0.0674; day 4, p = 0.7046. (C, F) Dynamic changes in microglial cell numbers (green) and corresponding normalized calcium signal intensity (red) within the 120 µm radius surrounding the graft site over 5 days. Differences were analyzed by two-way ANOVA followed by Tukey’s test. (* *p <* 0.05, ** *p <* 0.01, **** *p <* 0.0001).

**Supplementary Table 1. Literature survey of cell concentrations for intracerebral injection.**

| **Concentration (cells/μl)** | **Year** | **Cell type** | **Reference** |
| --- | --- | --- | --- |
| 100000 | 2006 | mesencephalon-derived hNPCs | Hovakimyan, M., et al. [1] |
| 200000 | 2009 | hNPC | Hicks, A.U., et al. [2] |
| 4000; 20000 | 2011 | NPC and BMSC | Kondziolka, D., et al. [3] |
| 100000 | 2012 | human iPSC-derived | Oki, K., et al. [4] |
| 20000; 40000 | 2012 | adipose-derived stem cells | Chen, J., et al. [5] |
| 100000 | 2013 | hiPSC-NSCs | Tornero, D., et al. [6] |
| 100000 | 2013 | C17.2 | Liang, Y., et al. [7] |
| 20000 | 2018 | Bone-derived MSCs | Cheng, Z., et al. [8] |
| 30000 | 2018 | Human cortex-derived NSCs | McGinley, L.M., et al. [9] |
| 50000 | 2018 | hPESC-derived DA neurons | Wang, Y.K., et al. [10] |
| 100000 | 2019 | MSCs | Peruzzaro, S.T., et al. [11] |
| 75000; 37500 | 2019 | hESC-derived VM-DA progenitors | Adler, A.F., et al. [12] |
| 100000 | 2020 | Directly Reprogrammed Human Neural Precursor Cells | Vonderwalde, I. [13] |
| 2000; 20000; 200000 | 2020 | human adipose-derived stem cell | Chang, C., et al. [14] |
| 80000 | 2021 | hNSCs (line HK532-CAG-IGF1) | McGinley, L.M. [15] |
| 80000 | 2023 | SB623 cells | Yabuno, S., et al. [16] |
| 20000; 100000 | 2023 | h-iSCs | Nakagomi, T., et al. [17] |
| 100000 | 2023 | hPSC-derived long-term expandable NES cells | Zhuang, Z., et al.[18] |
| 50000 | 2023 | hiPSC-NPCs | Kanemura, Y., et al. [19] |
| 60000 | 2024 | hematopoietic stem/progenitor cells | Milazzo, R., et al. [20] |
| 20000 | 2024 | hNSCs | Liu, Y.F., et al. [21] |
| 100000 | 2024 | h-brain-derived iSCs / h-MSCs | Tanada, S., et al. [22] |
| 100000 | 2024 | P19 ECCs | Amini, A et al. [23] |
| 100000 | 2025 | hiPSC-NPCs | Weber, R.Z., et al. [24] |
| 100000 | 2025 | NPC | Weber, R.Z., et al. [25] |
| 67000; 170000; 330000 | 2025 | Autologous MSCs | Myers, M.I., et al. [26] |

**Reference**

1. Hovakimyan, M., et al., *Mesencephalic human neural progenitor cells transplanted into the neonatal hemiparkinsonian rat striatum differentiate into neurons and improve motor behaviour.* J Anat, 2006. **209**(6): p. 721-32.

2. Hicks, A.U., et al., *Transplantation of human embryonic stem cell-derived neural precursor cells and enriched environment after cortical stroke in rats: cell survival and functional recovery.* Eur J Neurosci, 2009. **29**(3): p. 562-74.

3. Kondziolka, D., et al., *Injection parameters affect cell viability and implant volumes in automated cell delivery for the brain.* Cell Transplant, 2011. **20**(11-12): p. 1901-6.

4. Oki, K., et al., *Human-induced pluripotent stem cells form functional neurons and improve recovery after grafting in stroke-damaged brain.* Stem Cells, 2012. **30**(6): p. 1120-33.

5. Chen, J., et al., *Transplantation of adipose-derived stem cells is associated with neural differentiation and functional improvement in a rat model of intracerebral hemorrhage.* CNS Neurosci Ther, 2012. **18**(10): p. 847-54.

6. Tornero, D., et al., *Human induced pluripotent stem cell-derived cortical neurons integrate in stroke-injured cortex and improve functional recovery.* Brain, 2013. **136**(Pt 12): p. 3561-77.

7. Liang, Y., et al., *Neural progenitor cell survival in mouse brain can be improved by co-transplantation of helper cells expressing bFGF under doxycycline control.* Exp Neurol, 2013. **247**: p. 73-9.

8. Cheng, Z., et al., *Mesenchymal stem cells attenuate blood-brain barrier leakage after cerebral ischemia in mice.* J Neuroinflammation, 2018. **15**(1): p. 135.

9. McGinley, L.M., et al., *Human neural stem cell transplantation improves cognition in a murine model of Alzheimer's disease.* Sci Rep, 2018. **8**(1): p. 14776.

10. Wang, Y.K., et al., *Human Clinical-Grade Parthenogenetic ESC-Derived Dopaminergic Neurons Recover Locomotive Defects of Nonhuman Primate Models of Parkinson's Disease.* Stem Cell Reports, 2018. **11**(1): p. 171-182.

11. Peruzzaro, S.T., et al., *Transplantation of mesenchymal stem cells genetically engineered to overexpress interleukin-10 promotes alternative inflammatory response in rat model of traumatic brain injury.* J Neuroinflammation, 2019. **16**(1): p. 2.

12. Adler, A.F., et al., *hESC-Derived Dopaminergic Transplants Integrate into Basal Ganglia Circuitry in a Preclinical Model of Parkinson's Disease.* Cell Rep, 2019. **28**(13): p. 3462-3473.e5.

13. Vonderwalde, I., et al., *Transplantation of Directly Reprogrammed Human Neural Precursor Cells Following Stroke Promotes Synaptogenesis and Functional Recovery.* Transl Stroke Res, 2020. **11**(1): p. 93-107.

14. Chang, C., et al., *Transplantation of Adipose-Derived Stem Cells Alleviates Striatal Degeneration in a Transgenic Mouse Model for Multiple System Atrophy.* Cell Transplant, 2020. **29**: p. 963689720960185.

15. McGinley, L.M., et al., *Magnetic resonance imaging of human neural stem cells in rodent and primate brain.* Stem Cells Transl Med, 2021. **10**(1): p. 83-97.

16. Yabuno, S., et al., *Synergistic therapeutic effects of intracerebral transplantation of human modified bone marrow-derived stromal cells (SB623) and voluntary exercise with running wheel in a rat model of ischemic stroke.* Stem Cell Res Ther, 2023. **14**(1): p. 10.

17. Nakagomi, T., et al., *Transplantation of Human Brain-Derived Ischemia-Induced Multipotent Stem Cells Ameliorates Neurological Dysfunction in Mice After Stroke.* Stem Cells Transl Med, 2023. **12**(6): p. 400-414.

18. Park, T.Y., et al., *Co-transplantation of autologous T(reg) cells in a cell therapy for Parkinson's disease.* Nature, 2023. **619**(7970): p. 606-615.

19. Kanemura, Y., et al., *Human-Induced Pluripotent Stem Cell-Derived Neural Progenitor Cells Showed Neuronal Differentiation, Neurite Extension, and Formation of Synaptic Structures in Rodent Ischemic Stroke Brains.* Cells, 2024. **13**(8).

20. Milazzo, R., et al., *Therapeutic efficacy of intracerebral hematopoietic stem cell gene therapy in an Alzheimer's disease mouse model.* Nat Commun, 2024. **15**(1): p. 8024.

21. Liu, Y.F., et al., *Stereotactically intracerebral transplantation of neural stem cells for ischemic stroke attenuated inflammatory responses and promoted neurogenesis: an experimental study with monkeys.* Int J Surg, 2024. **110**(9): p. 5417-5433.

22. Tanada, S., et al., *Human-Brain-Derived Ischemia-Induced Stem Cell Transplantation Is Associated with a Greater Neurological Functional Improvement Compared with Human-Bone Marrow-Derived Mesenchymal Stem Cell Transplantation in Mice After Stroke.* Int J Mol Sci, 2024. **25**(22).

23. Amini, A., F. Esmaeili, and M. Golpich, *Possible role of lncRNAs in amelioration of Parkinson's disease symptoms by transplantation of dopaminergic cells.* NPJ Parkinsons Dis, 2024. **10**(1): p. 56.

24. Weber, R.Z., et al., *Neural xenografts contribute to long-term recovery in stroke via molecular graft-host crosstalk.* Nat Commun, 2025. **16**(1): p. 8224.

25. Weber, R.Z., et al., *Delayed Transplantation of Neural Stem Cells Improves Initial Graft Survival after Stroke.* Adv Sci (Weinh), 2025. **12**(29): p. e04154.

26. Myers, M.I., et al., *Intracerebral Transplantation of Autologous Mesenchymal Stem Cells Improves Functional Recovery in a Rat Model of Chronic Ischemic Stroke.* Transl Stroke Res, 2025. **16**(2): p. 248-261.

**Supplemental python codes for modeling**

**Figure 3B Simulated cell retention rate at different beta value.**


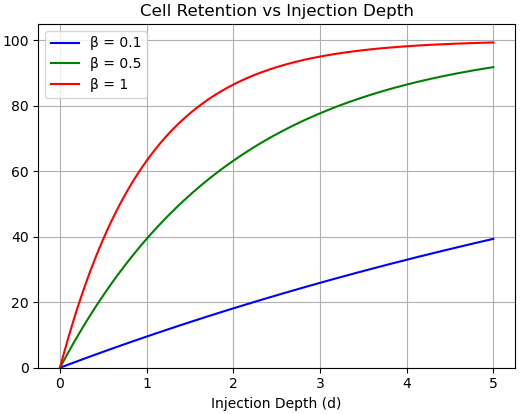


import numpy as np

import matplotlib.pyplot as plt

# Define the simplified retention function

def R(d, beta):

return 100 * (1 - np.exp(-beta * d))

# Depth values from 0 to 5 mm

d_values = np.linspace(0, 5, 200)

# Try different beta values to see the effect

beta_values = [0.1, 0.5, 1, 2]

colors = ['blue', 'green', 'red']

plt.figure(figsize=(8, 5))

for beta, color in zip(beta_values, colors):

R_values = R(d_values, beta)

plt.plot(d_values, R_values, label=f'β = {beta}', color=color)

plt.title("Cell Retention vs Injection Depth")

plt.xlabel("Injection Depth (d)")

plt.ylabel("Retention Rate (%)")

plt.ylim(0, 105)

plt.grid(True)

plt.legend()

plt.tight_layout()

plt.show()

**Figure 3F Fitting the theoretical curve with experimental data from Figure 3E**


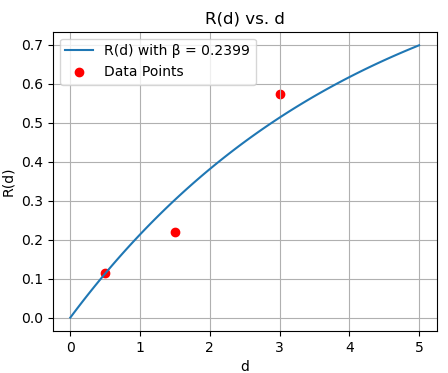


import numpy as np

import matplotlib.pyplot as plt

from scipy.optimize import minimize

# Define the function R(d)

def r_function(d, beta):

return 1 - np.exp(-beta * d)

# Given data points

data_points = [

(3, 0.573), # R = 57.3% = 0.573

(1.5, 0.2186), # R = 21.86% = 0.2186

(0.5, 0.1134) # R = 11.34% = 0.1134

]

# Define the error function to minimize

def error_function(beta, data_points):

error = 0

for d, r in data_points:

r_predicted = r_function(d, beta)

error += (r_predicted - r) ** 2 # Sum of squared errors

return error

# Initial guess for beta

initial_beta = 0.1

# Use scipy.optimize.minimize to find the optimal beta

result = minimize(error_function, initial_beta, args=(data_points,))

optimal_beta = result.x[0]

# Calculate R-squared

r_values = np.array([r for _, r in data_points])

r_predicted_values = r_function(np.array([d for d, _ in data_points]), optimal_beta)

mean_r = np.mean(r_values)

ss_total = np.sum((r_values - mean_r) ** 2)

ss_residual = np.sum((r_values - r_predicted_values) ** 2)

r_squared = 1 - (ss_residual / ss_total)

# Print the optimal beta and R-squared

print(f"Optimal beta: {optimal_beta:.4f}")

print(f"R-squared: {r_squared:.4f}")

# Generate a range of d values for plotting

d_values = np.linspace(0, 5, 100)

r_values_plot = r_function(d_values, optimal_beta)

# Plot the function R(d) with the optimal beta

plt.figure(figsize=(8, 6))

plt.plot(d_values, r_values_plot, label=f'R(d) with β = {optimal_beta:.4f}')

plt.scatter([d for d, _ in data_points], [r for _, r in data_points], color='red', label='Data Points')

plt.xlabel('d')

plt.ylabel('R(d)')

plt.title('R(d) vs. d')

plt.grid(True)

plt.legend()

plt.show()

**Supplementary Figure 2. The mono-exponential decay model for evaluating oxygen diffusion in tissue.**


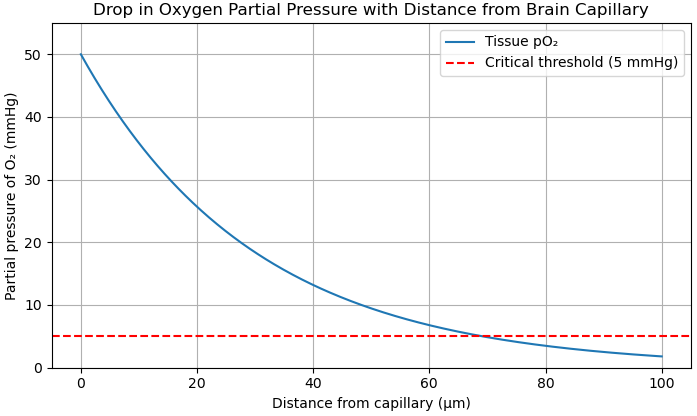


import numpy as np

import matplotlib.pyplot as plt

# Simulate distance from a capillary (in micrometers)

distance_um = np.linspace(0, 100, 500) # 0 to 100 µm

# Model: exponential decay of pO2 with distance from capillary

# Typical capillary pO2 ~50 mmHg, drops to ~5 mmHg at ~100 µm

pO2 = 50 * np.exp(-distance_um / 30) # 30 µm decay length approximates tissue consumption

# Plot

# Re-plot with y-axis starting from 0

plt.figure(figsize=(8, 5))

plt.plot(distance_um, pO2, label='Tissue pO₂')

plt.axhline(5, color='red', linestyle='--', label='Critical threshold (5 mmHg)')

plt.xlabel('Distance from capillary (µm)')

plt.ylabel('Partial pressure of O₂ (mmHg)')

plt.title('Drop in Oxygen Partial Pressure with Distance from Brain Capillary')

plt.ylim(0, 55)

plt.grid(True)

plt.legend()

plt.tight_layout()

plt.show()

**Figure 3I HypoR as a function of radius of implanted cell mass.**


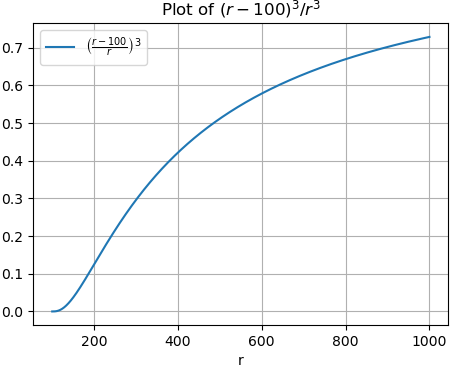


import matplotlib.pyplot as plt

import numpy as np

# Define the range of r

r = np.linspace(100, 1000, 1000)

# Compute the function

y = ((r - 100)**3) / (r**3)

# Plotting

plt.figure(figsize=(8, 5))

plt.plot(r, y, label=r'$\left(\frac{r - 100}{r}\right)^3$')

plt.xlabel('r')

plt.ylabel('Function Value')

plt.title('Plot of $(r - 100)^3 / r^3$')

plt.grid(True)

plt.legend()

plt.tight_layout()

plt.show()

**Supplementary Figure 3. HypoR as a function of implanted cell volume.**


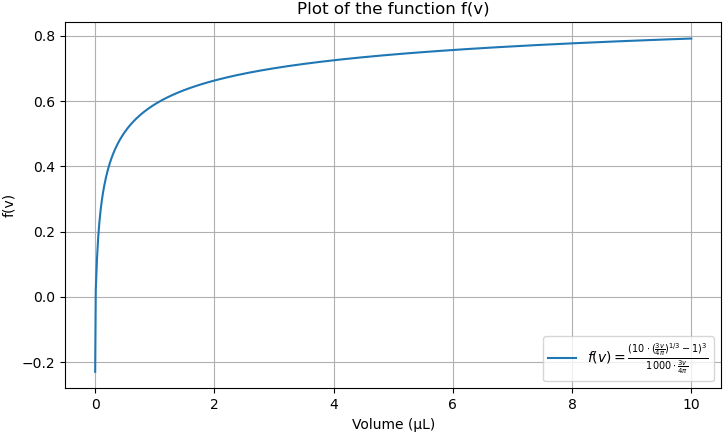


import numpy as np

import matplotlib.pyplot as plt

# Volume range in microliters

v = np.linspace(0.001, 10, 1000)

# Compute the function

numerator = (10 * (3 * v / (4 * np.pi))**(1/3) - 1)**3

denominator = 1000 * (3 * v / (4 * np.pi))

f_v = numerator / denominator

# Plotting

plt.figure(figsize=(8, 5))

plt.plot(v, f_v, label=r'$f(v) = \frac{(10 \cdot (\frac{3v}{4\pi})^{1/3} - 1)^3}{1000 \cdot \frac{3v}{4\pi}}$')

plt.xlabel('Volume (µL)')

plt.ylabel('f(v)')

plt.title('Plot of the function f(v)')

plt.grid(True)

plt.legend()

plt.tight_layout()

plt.show()

**Figure 3M Fitting the theoretical curve with experimental data from Fig. 3L.**


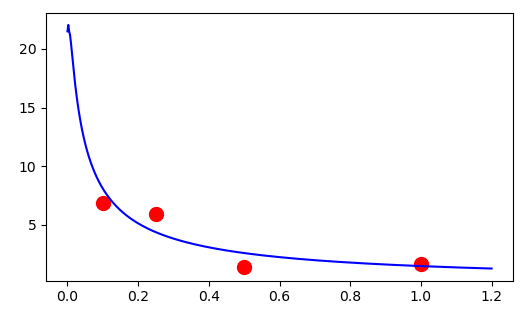


import numpy as np

import matplotlib.pyplot as plt

# Fixed gmax value

gmax_fixed = 21.5

# Actual data points

v_data = np.array([0.1, 0.25, 0.5, 1.0])

G_data = np.array([6.88, 5.9, 1.44, 1.64])

# Define the term function

def calculate_term(v):

    return (3 * v / (4 * np.pi))**(1/3)

# Define the function f_v (Corrected to handle array division)

def calculate_fv(v):

    term = calculate_term(v)

    numerator = (10 * term - 1)**3

    denominator = 1000 * (3 * v / (4 * np.pi))

    # Avoid division by zero for v=0 using np.where

    return np.where(denominator != 0, numerator / denominator, 0)

# Define the Growth rate model G = gmax * (1 - f_v)**3

def growth_rate_model(v, gmax):

    fv = calculate_fv(v)

    return gmax * (1 - fv)**3

# Generate v values for plotting the fitted curve

v_fit = np.linspace(0, 1.2, 500) # Extend the range slightly to see the curve

# Calculate the fitted G values using the fixed gmax

G_fit = growth_rate_model(v_fit, gmax_fixed)

# Calculate the model values at the data points using the fixed gmax

G_model_at_data = growth_rate_model(v_data, gmax_fixed)

# Calculate residuals

residuals = G_data - G_model_at_data

# Calculate R-squared

ss_res = np.sum(residuals**2)

ss_tot = np.sum((G_data - np.mean(G_data))**2)

r_squared = 1 - (ss_res / ss_tot)

print(f"R-squared (with fixed gmax={gmax_fixed}): {r_squared:.4f}")

# Plot the data and the fitted model

plt.figure(figsize=(10, 6))

plt.scatter(v_data, G_data, label='Actual Data', color='red', marker='o', s=100)

plt.plot(v_fit, G_fit, label=f'Model (gmax={gmax_fixed})', color='blue')

# Plot residuals

plt.figure(figsize=(10, 6))

plt.scatter(v_data, residuals, color='green', marker='x', s=100, label='Residuals')

plt.axhline(y=0, color='black', linestyle='--')

plt.xlabel('v')

plt.ylabel('Residuals (G_data - G_model)')

plt.title('Residual Plot')

plt.grid(True)

plt.legend()

plt.show()

plt.figure(figsize=(10, 6))

plt.scatter(v_data, G_data, label='Actual Data', color='red', marker='o', s=100)

plt.plot(v_fit, G_fit, label=f'Model (gmax={gmax_fixed})', color='blue')

plt.xlabel('v')

plt.ylabel('Growth Rate (G)')

plt.title('Fitting the Growth Rate Model to Actual Data')

plt.grid(True)

plt.legend()

plt.ylim(0, 7.5)

plt.xlim(0, 1.2)

plt.show()

**Supplementary Figure 5. Fitting experimental data to formula with different exponential.**


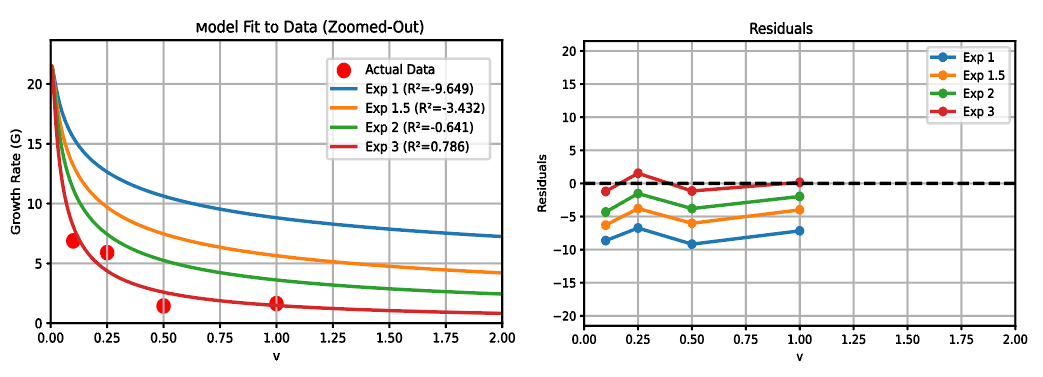


import numpy as np

import matplotlib.pyplot as plt

# Constants and data

gmax_fixed = 21.5

v_data = np.array([0.1, 0.25, 0.5, 1.0])

G_data = np.array([6.88, 5.9, 1.44, 1.64])

exponents = [1, 1.5, 2, 3]

#exponents = [3]

# Define f_v calculation

def calculate_term(v):

    return (3 * v / (4 * np.pi))**(1/3)

def calculate_fv(v):

    term = calculate_term(v)

    numerator = (10 * term - 1)**3

    denominator = 1000 * (3 * v / (4 * np.pi))

    return np.where(denominator != 0, numerator / denominator, 0)

# Define G = gmax * (1 - f_v)^exponent

def growth_rate_model(v, gmax, exponent):

    fv = calculate_fv(v)

    return gmax*(1 - fv)**exponent

# v values for smooth plot

v_fit = np.linspace(0, 2.0, 500)

# ---- Figure 1: Model Fit (Zoomed-in) ----

plt.figure(figsize=(10, 6))

plt.scatter(v_data, G_data, color='red', s=100, label='Actual Data')

for i, exp in enumerate(exponents):

    G_fit = growth_rate_model(v_fit, gmax_fixed, exp)

    G_model_at_data = growth_rate_model(v_data, gmax_fixed, exp)

    residuals = G_data - G_model_at_data

     # R-squared

    ss_res = np.sum(residuals**2)

    ss_tot = np.sum((G_data - np.mean(G_data))**2)

    r_squared = 1 - ss_res / ss_tot

    label = f'Exp {exp} (R²={r_squared:.3f})'

    plt.plot(v_fit, G_fit, label=label)

     # Optional: Add text box with R²

    plt.text(1.25, 7 - i*0.5, f'Exp {exp}: R² = {r_squared:.3f}', fontsize=10)

plt.xlabel('v')

plt.ylabel('Growth Rate (G)')

plt.title('Figure 1: Model Fit to Data (Zoomed-In)')

plt.legend()

plt.grid(True)

plt.ylim(0, 7.5)

plt.xlim(0, 1.2)

plt.tight_layout()

plt.show()

# ---- Figure 2: Model Fit (Zoomed-out) ----

plt.figure(figsize=(10, 6))

plt.scatter(v_data, G_data, color='red', s=100, label='Actual Data')

for i, exp in enumerate(exponents):

    G_fit = growth_rate_model(v_fit, gmax_fixed, exp)

    G_model_at_data = growth_rate_model(v_data, gmax_fixed, exp)

    residuals = G_data - G_model_at_data

    ss_res = np.sum(residuals**2)

    ss_tot = np.sum((G_data - np.mean(G_data))**2)

    r_squared = 1 - ss_res / ss_tot

    label = f'Exp {exp} (R²={r_squared:.3f})'

    plt.plot(v_fit, G_fit, label=label)

    plt.text(1.5, gmax_fixed - i*1.2, f'Exp {exp}: R² = {r_squared:.3f}', fontsize=10)

    plt.xlabel('v')

plt.ylabel('Growth Rate (G)')

plt.title('Figure 2: Model Fit to Data (Zoomed-Out)')

plt.legend()

plt.grid(True)

plt.ylim(0, gmax_fixed * 1.1)

plt.xlim(0, 2.0)

plt.tight_layout()

plt.show()

    # ---- Figure 3: Residuals (Zoomed-in) ----

plt.figure(figsize=(10, 6))

for exp in exponents:

    G_model_at_data = growth_rate_model(v_data, gmax_fixed, exp)

    residuals = G_data - G_model_at_data

    plt.plot(v_data, residuals, marker='o', label=f'Exp {exp}')

    plt.axhline(0, color='black', linestyle='--')

plt.xlabel('v')

plt.ylabel('Residuals')

plt.title('Figure 3: Residuals (Zoomed-In)')

plt.grid(True)

plt.legend()

plt.ylim(-2, 2)

plt.xlim(0, 1.2)

plt.tight_layout()

plt.show()

    # ---- Figure 4: Residuals (Zoomed-out) ----

plt.figure(figsize=(10, 6))

for exp in exponents:

    G_model_at_data = growth_rate_model(v_data, gmax_fixed, exp)

    residuals = G_data - G_model_at_data

    plt.plot(v_data, residuals, marker='o', label=f'Exp {exp}')

    plt.axhline(0, color='black', linestyle='--')

plt.xlabel('v')

plt.ylabel('Residuals')

plt.title('Figure 4: Residuals (Zoomed-Out)')

plt.grid(True)

plt.legend()

plt.ylim(-gmax_fixed, gmax_fixed)

plt.xlim(0, 2.0)

plt.tight_layout()

plt.show()
